# Ethoxy Meso-Modified Heptamethine Cyanine Fluorophores: Synthesis, Photophysical Properties and BSA Sensing Study

**DOI:** 10.3390/molecules31132267

**Published:** 2026-06-29

**Authors:** Tarek Erfan Ahmed, Maged Henary

**Affiliations:** Department of Chemistry and Center for Diagnostics and Therapeutics, Georgia State University, Atlanta, GA 30303, USA; tahmed13@student.gsu.edu

**Keywords:** NIR fluorophores, Heptamethine cyanine, meso substitution, bovine serum albumin, turn-on fluorescence

## Abstract

Heptamethine cyanine fluorophores are widely used in NIR imaging, due to their excellent photophysical properties. In the present work, new meso-ethoxy substituted heptamethine carbocyanine fluorophores **6a–c** were prepared by replacing the meso-chlorine atom on a cyclohexenyl-bridged scaffold through a base-driven S_RN_1 substitution reaction with an ethoxy group. Once isolated, the compounds were examined in several solvents to study how strongly the medium influenced their absorption, emission, and brightness. The fluorophores absorbed in the 755–770 nm range and showed their strongest fluorescence in ethanol, whereas their emissions were heavily quenched in buffer solutions. Calculations of the HOMO and LUMO energies supported the observed data. A comparison with indocyanine green (ICG) under continuous light exposure showed that fluorophores **6a–c** degraded more slowly and maintained their absorbance for a considerably longer period. Having long hydrophobic alkyl chains raised the hypothesis that they could act as sensors for bovine serum albumin (BSA), which is known for its hydrophobic pockets. Therefore, they were tested for BSA binding both in silico and in cuvette. Fluorophore **6b** produced a clear optical response when BSA was added, giving both a red shift and an increase in absorbance and fluorescence. Quantitative analysis of the fluorophore **6b**–BSA interaction revealed a dissociation constant (K_d_) of 0.75 μM and an apparent binding stoichiometry of approximately 1:1 (Hill coefficient *n* = 1.25), confirming high-affinity, single-site binding. Using the linear range of the fluorescence titration, the limit of detection (LOD) and limit of quantitation (LOQ) for BSA were determined to be 1.9 and 6.0 μM, respectively. Selectivity experiments demonstrated that fluorophore **6b** does not exhibit fluorescence enhancement in the presence of collagen or human parvalbumin, confirming its selectivity toward BSA. These observations suggest that introducing an alkoxy group at the meso-position can improve carbocyanine fluorophores properties and achieve high photostability, with significant potential for various biomedical applications.

## 1. Introduction

Polymethine cyanine fluorophores remain one of the most important families of NIR fluorophores because of their large molar absorptivity and the versatility of their structures for modifications [1,2,3,4,5]. Their extended π-conjugation lowers the HOMO–LUMO gap enough to push their optical transitions deep into the NIR region [6,7,8]. However, the widely used clinical dye indocyanine green (ICG) shows several drawbacks; its moderate quantum yield, limited photostability, strong aggregation in physiological media, and lack of straightforward bioconjugation sites all restrict its broader use [9,10,11]. These shortcomings continue to motivate researchers to develop new cyanine dyes that preserve favorable NIR features, while offering higher brightness, improved resistance to degradation, and more selective biological targeting for imaging and surgical guidance [12,13,14,15].

A common strategy to stabilize cyanine fluorophores involves incorporating a rigid cyclohexenyl ring into the heptamethine chain [16,17,18]. This added rigidity limits non-radiative decay processes, such as cis–trans-photoisomerization, and contributes to better quantum yields and photostability. The cyclohexenyl ring also provides a practical handle for chemical modification at the electron-poor meso-carbon, which typically carries a reactive chlorine atom [16]. This meso-chlorine is particularly useful because it readily undergoes radical-nucleophilic aromatic substitution (S_RN_1), allowing a wide range of functional groups, including amines and other polar substituents, to be introduced. Such modifications make it possible to tune solubility, aggregation tendencies, and absorption properties to better suit deep-tissue imaging applications [13,19,20].

Among the various meso-substitutions explored, alkoxy groups (O-alkyl substituents) offer a highly stable alternative [21,22]. The resulting C–O ether bond is much more resistant to hydrolysis than the meso-chlorine, and the alkoxy group primarily exerts an inductive effect rather than strong electronic donation [23,24]. This tends to produce a small blue shift and improved stability [25]. Removing the chlorine atom also reduces the likelihood of unwanted reactions with biological nucleophiles such as cysteine or glutathione, which can decrease cytotoxicity and enhance the overall in vivo behavior of the dye [3,13,16,26].

Serum albumin is a key biomarker in diagnostics, and many cyanine dyes exhibit affinity for its hydrophobic binding pockets [27,28,29]. Their interaction typically produces a fluorescence “turn-on” effect [29,30]. In aqueous buffer, cyanines often form non-emissive H-aggregates; binding to albumin disrupts these aggregates and isolates the dye molecules within the protein, restoring the bright monomeric emission [28,30]. Effective probe design requires balancing the dye’s natural tendency to aggregate with its ability to bind strongly to albumin, ensuring a large turn-on response [31,32].

In this context, we report the design and synthesis of fluorophores **6a–c** as new heptamethine carbocyanine fluorophores bearing a meso-ethoxy substituent. Replacing the reactive meso-chlorine atom with a stable ether was expected to improve both chemical and photothermal stability while optimizing interactions with bovine serum albumin (BSA). This study combined a base-mediated synthetic approach with detailed photophysical measurements, such as solvatochromic behavior, and density functional theory (DFT) calculations to clarify the electronic and structural features of the dyes. Their stability was directly compared to ICG, and the performance of fluorophore **6b** as a fluorogenic probe for BSA was evaluated in terms of sensitivity, dynamic range, and the origin of the fluorescence enhancement. Together, these investigations aimed to establish meso-alkoxy carbocyanines as promising candidates for next-generation optical probes.

## 2. Results and Discussion

### 2.1. Chemistry

**Scheme 1:**
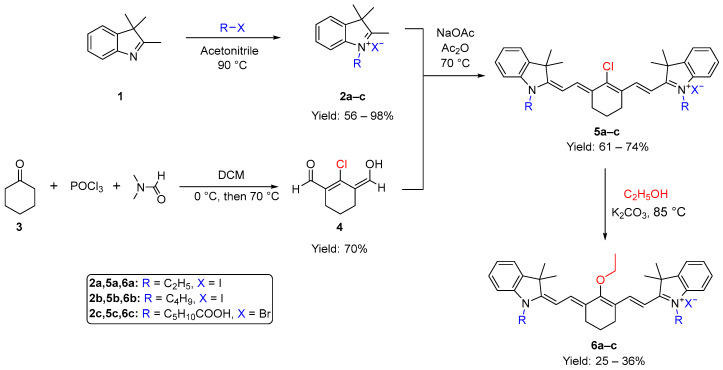
Synthesis of the heptamethine cyanine fluorophores **6a–c**.

Fluorophores **6a–c** were synthesized according to our reported procedure [2,7,33,34,35,36]. Mainly, the synthetic scheme (Scheme 1) started with the alkylation of 2,3,3- trimethylindolenine **1** by various alkyl halides to form the indolium salts **2a–c**. In parallel, Vilsmeier Chloroformylation of cyclohexanone **3** was done using phosphorus oxychloride and dimethylformamide to form the dialdehyde linker **4**. Then, condensation of the indolium salts **2a–c** with the linker **4** yielded the heptamethine carbocyanine dyes **5a–c** with the meso-chlorine atom.

The next step was the meso-substitution of the heptamethine dye **5a–c** with ethanol. The reaction mixture involved using ethanol as the solvent and the reagent, and potassium carbonate as the base for the meso-substitution. Potassium carbonate deprotonated the ethanol forming the ethoxide ion, which attacked the carbon atom at the meso-position and substituted the chlorine atom through S_RN_1 mechanism to form the ethoxy-substituted heptamethine cyanine fluorophores **6a–c**.

**Scheme 2:**
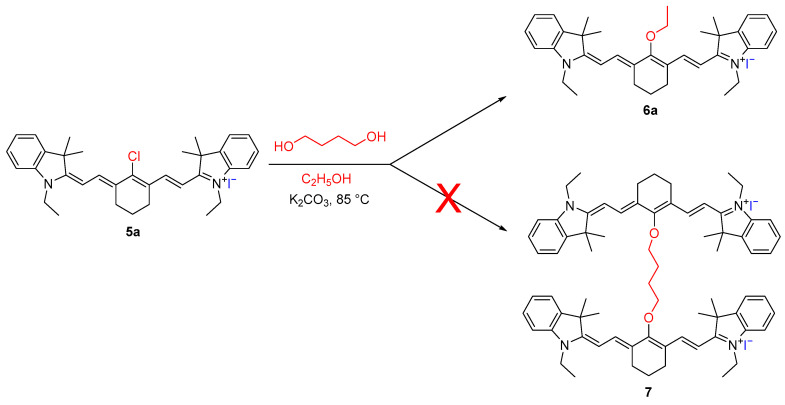
Actual vs. expected routes for the reaction of fluorophore **5a** with 1,4-butanediol in the presence of ethanol as the solvent.

Fluorophore **6a** was prepared before by Miltsov et al. as an intermediate, so it was not characterized or studied photophysically [37]. Interestingly, fluorophore **6a** was first prepared in a trial to synthesize dimeric heptamethine cyanine fluorophores as a part of a previously published project, in which we synthesized a series of dimeric heptamethine fluorophores using various nitrogen containing linkers [3]. The heptamethine dye **5a** was reacted with 1,4-butanediol in the presence of ethanol as the solvent and potassium carbonate as the base. It was expected that the 1,4-butanediol oxygen atoms would replace the meso-chlorine atoms in two molecules of the monomeric dye and form the dimeric heptamethine dye, but fluorophore **6a** was produced instead (Scheme 2).

This was explained by the fact that both of the oxygen atoms of ethanol and 1,4-butanediol possessed comparable nucleophilicity, but ethanol existed in the reaction mixture in a much higher concentration than 1,4-butanediol (excess amount vs. 1 equivalent); therefore, the reaction with ethanol had higher probability to occur than the reaction with 1,4-butanediol. In addition, ethanol is a smaller molecule than 1,4-butanediol, so it was easier to react. Finally, the dimer formation reaction was found to require harsh conditions and a long amount of time; that is why the reaction with ethanol was more preferred [3]. The formation of fluorophore **6a** was confirmed by performing high-resolution mass spectroscopy analysis of the product of this reaction, and it was found that the molecular ion peak for the expected dimeric dye was absent. Instead, a major peak at *m*/*z* 521.35 was found, which was attributed to the formation of fluorophore **6a**. Furthermore, a Correlation Spectroscopy (COSY) 2D NMR experiment was carried out on compound **6a**, along with the routine ^1^HNMR and^13^CNMR, and all experiments confirmed the preferential formation of the ethoxy meso-modified fluorophore **6a**.

### 2.2. Photophysical Properties

The photophysical properties of fluorophores **6a–c** were studied in different solvents, which were ethanol, DMSO, 4-(2-hydroxyethyl)-1-piperazineethanesulfonic acid (HEPES) buffer, and phosphate-buffered saline (PBS), and compared to the FDA-approved indocyanine green (ICG) (Figure S13) as shown in Table 1 and Figures S14–S16 [9,10]. Ethanol, a polar protic solvent, and DMSO, a polar aprotic solvent, were used as examples of organic solvents to understand the physicochemical behavior of the fluorophores. The buffers used were aqueous-based to understand the behavior of the fluorophores in biologically relevant solvents [38,39]. HEPES buffer was used in a concentration of 50 mM and has a pH of 7.4, and PBS was used in a concentration of 1X PBS, with a pH of 7.4 as well to match the physiological pH.

The absorbance wavelength maxima ranged from 755 to 770 nm in different solvents, with a little bathochromic shift in DMSO. The emission wavelength maxima spanned from 768 to 791 nm, with the same trend as the absorption. Fluorophore **6c** possessed the longest emission maximum in DMSO, and fluorophore **6a** had the shortest emission maxima in PBS buffer. The Stokes shift was highest in HEPES buffer (21 nm) for **6b** and in DMSO (21 nm) for **6c** and lowest in PBS buffer (13 nm) for **6a**, with a small variation between the different solvents. The extinction coefficient was highest in ethanol, followed by DMSO, with the largest value for fluorophore **6b** (214,600 M^−1^cm^−1^ in ethanol). It was usually lower in the buffer solutions due to the lower solubility or aggregation, with the lowest value for fluorophore **6c** in HEPES buffer (27,400 M^−1^cm^−1^). Fluorophore **6c** consistently possessed lower extinction coefficient compared to the other fluorophores, due to the presence of carboxylic groups with their increased ability to make hydrogen bonds. Stronger hydrogen bonding drives aggregation, which reduces the extinction coefficient through altered electronic environments and energy transfer processes [40]. Additionally, interactions with polar solvents likely disrupt molecular planarity, thereby diminishing conjugation [41]. Overall, the extinction coefficient values for fluorophores **6a** and **6b** were comparable to ICG in the different solvents.

The quantum yield of fluorescence was calculated as well and had values between 0.357% and 17.3%. For fluorophore **6b**, it was highest in ethanol (17.3%), which was higher than ICG as well. However, it was much lower in the buffer solutions, especially HEPES buffer, with a value of 0.418%. The values of the quantum yield of fluorescence were comparable to ICG in ethanol and DMSO but were much lower in the buffer solutions, presumably due to the variation in aqueous solubility between the two fluorophores [42,43]. Fluorophore **6b** had much lower solubility in the buffer solutions because of its highly hydrophobic butyl groups. A similar trend for the quantum yield was found for fluorophore **6a**, with a slightly higher value in PBS buffer, due to the less hydrophobic ethyl groups compared to the butyl groups. Fluorophore **6c** was slightly different, as the value was the highest in DMSO (13.74%), and its values in the buffer solutions were not very low and comparable to ICG, likely due to the lack of solubility issues that fluorophores **6a** and **6b** suffered.

The molecular brightness is calculated by multiplying the extinction coefficient and quantum yield of fluorescence, and it is indicative of how bright the molecule is when irradiated by light [44,45]. The values for fluorophore **6b** ranged from 37,437 M^−1^cm^−1^ in ethanol to 736.5 M^−1^cm^−1^ in HEPES buffer, showing the same trend as the quantum yield of fluorescence. Compared to ICG, the molecular brightness of fluorophore **6b** was higher in ethanol, and lower in all other solvents, apparently due to the solubility differences [46]. Fluorophore **6a** followed by **6c** had slightly lower values compared to **6b**, due to the lower extinction coefficients.

### 2.3. Photothermal Stability Study

**Figure 1:**
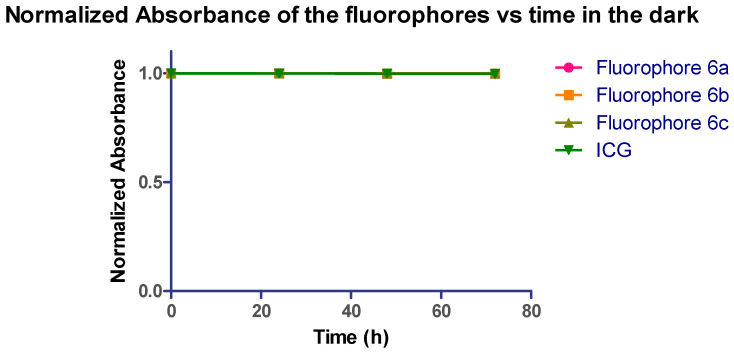
Photothermal stability of fluorophores **6a–c** and ICG (4 µM in ethanol); their normalized absorbance over time in the dark.

**Figure 2:**
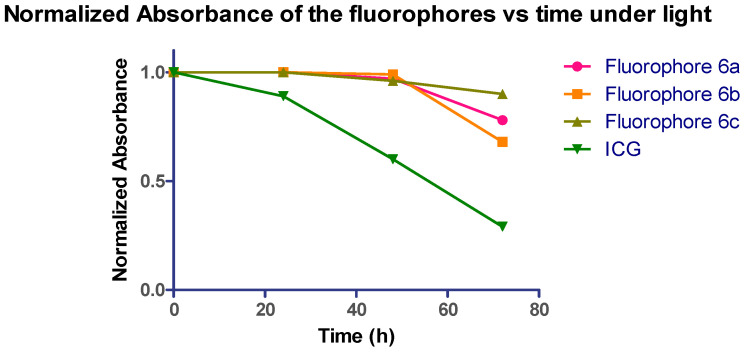
Photothermal stability of fluorophores **6a–c** and ICG (4 µM in ethanol); their normalized absorbance over time under irradiation with a 6000 mW 254 nm UV lamp placed 10 cm from the fluorophore’s solutions.

The stability of fluorophores **6a–c** toward light and heat was measured and compared to ICG in a photothermal stability study. The study encompassed using two sets of the fluorophores: one in dark conditions and the other irradiated continuously with a 6000 mW 254 nm UV lamp light placed 10 cm away from the fluorophore’s solutions. The absorbance of the fluorophores was measured before the test and then measured at different time intervals for the duration of 72 h (Figure 1 and Figure 2). All fluorophores were stable in the dark conditions, and their absorbance almost did not change for the total duration of the study including ICG.

Under the light conditions, however, the absorbance started to decrease for all fluorophores. Interestingly, fluorophores **6a–c** were more stable, and their absorbance almost did not change for 48 h but started to decrease after that. At 72 h, fluorophore **6a** reached 78% of its original absorbance, fluorophore **6b** possessed 68%, and fluorophore **6c** was the most stable, retaining 90% of its original absorbance. ICG, on the other hand, started decreasing gradually from the beginning, reaching 60% of its absorbance at 48 h and 29% at 72 h.

### 2.4. Physicochemical Properties

The physicochemical properties of fluorophores **6a–c** were predicted using Chemaxon^®^ Marvinsketch version 24.3.2 to enhance our understanding of their properties and interactions with their surrounding environment or biological molecules (Table 2) [47]. The distribution coefficient (Log D) was calculated, and its value ranged from 3.12 for **6c** to 7.00 for **6b**. This reflected the hydrophilic nature for **6c**, due to the two carboxylic acid groups, and the hydrophobic nature of **6b**, presumably due to the two hydrophobic butyl groups in its structure. The count of rotatable bonds was determined, and it varied from 7 (for fluorophore **6a**) to 17 (for fluorophore **6c**) according to the alkyl group attached to the nitrogen, where it increases with the size of the alkyl group. This metric is significant for understanding the molecule’s structural flexibility and its impact on charge transfer effectiveness [48,49,50].

Geometrical properties were calculated because they are crucial for describing a molecule, particularly in the context of its use as a fluorophore in biological studies [51,52]. The molecular volume was predicted, and its values also increased with the increase in the size of the alkyl group from 525.1 Å^3^ (for **6a**) to 683.2 Å^3^ (for **6c**). The surface area followed the same trend, and the total polar surface area (TPSA) was 15.48 Å^2^ for **6a** and **6b,** which was considered low, reflecting the relatively hydrophobic structure of the fluorophores; however, the TPSA was 90.08 Å^2^ for **6c** due to the added polarity from the carboxylic groups. Calculation of the hydrogen bond donors (HBD) and acceptors (HBA) was performed to evaluate the fluorophore’s capacity for hydrogen bonding, which governs its intermolecular interactions and its binding with biological molecules [26,53,54,55]. There were no hydrogen bond donors in the structure, and the hydrogen bond acceptor number was 2 for both **6a** and **6b**. In contrast, fluorophore **6c** had two hydrogen bond donors and six hydrogen bond acceptors due to the extra oxygen atoms it had. Polarizability measures how readily an external electric field can distort a molecule’s electron cloud. For fluorophores, this is a critical property because it determines their interaction with the surrounding environment, thereby affecting their spectral characteristics, such as their absorption and emission wavelengths [56]. The polarizability was 63.88 for **6a,** 71.27 for **6b**, and 79.53 for **6c**, demonstrating the difference in electronegativities in their structures.

### 2.5. DFT Studies

**Figure 3:**
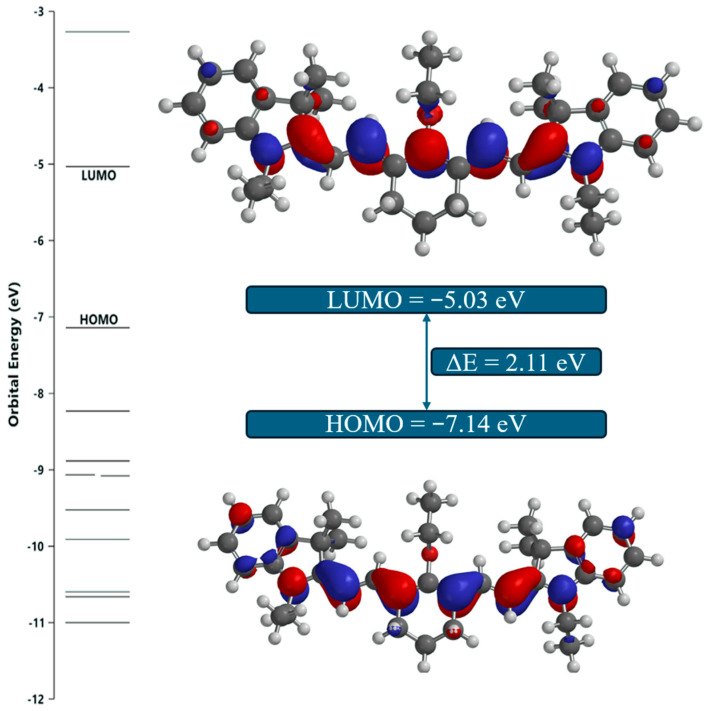
Frontier molecular orbitals (HOMO and LUMO) of fluorophore **6a** as predicted by the DFT studies with an energy gap of 2.11 eV.

Density functional theory (DFT) studies are essential tools for fluorophores to understand their fundamental electronic structures and predict their optical properties, such as absorption and emission wavelengths [57,58]. These calculations help rationalize experimental results and rationally design new fluorophore-based materials and probes with tailored performance for applications in biomedical imaging and sensing [59]. Therefore, density functional theory (DFT) studies were conducted for fluorophores **6a–c** to predict their most stable energy-preferred conformation and their frontier molecular orbitals (FMO) using Spartan^®^ 24 software (Figure 3, Figures S17 and S18) [60].

The most important FMOs were the highest occupied molecular orbital (HOMO) and the lowest occupied molecular orbital (LUMO), which determined the main energy gap responsible for the wavelength maxima of the fluorophores [61]. The predicted energy for the HOMO of fluorophore **6a** was −7.14 eV, and that for the LUMO was −5.03 eV, and by calculation, their energy gap was 2.11 eV. This energy gap can be used to calculate the expected absorption wavelength maxima using Planck–Einstein formula E = hc\λ, where E is the energy, h is Planck’s constant, c is the speed of light in a vacuum, and λ is the wavelength [62]. By substituting “hc” with 1240 eV. nm and E with 2.11 eV, the expected λ will be 587.7 nm, which is around 167 nm lower than the experimental absorption wavelength in ethanol. The observed blue shift between the calculated and experimental λ_max_ values is typical for DFT calculations, as it often overestimates excitation energies, struggles to correctly describe charge–transfer transitions, and can produce artificial low-energy states with very weak oscillator strengths [63,64]. Fluorophores **6b** and **6c** had very similar HOMO and LUMO energy predictions, due to the similarity in the structure and the pathway of the electron transfer between the three fluorophores (Figures S17 and S18). This was because they only differed in the alkyl group attached to the nitrogen atoms, which were away from the conjugated system where the electron transfer occurred.

### 2.6. Docking Study

Owing to the hydrophobic nature of the fluorophores manifested by their high log D values, and the long alkyl chains in their structures, it was hypothesized that these fluorophores could interact with bovine serum albumin (BSA) and bind in its hydrophobic pockets [27,28]. Guided by this hypothesis, computational docking was performed prior to the physical binding experiments to predict the binding affinity between the synthesized fluorophores and BSA. Because the exact binding sites were unidentified, a blind, global docking approach was executed using PyRx 0.8 software [65]. The BSA crystal structure (PDB ID: 4JK4) was obtained from the Research Collaboratory for Structural Bioinformatics (RCSB) Protein Data Bank [66]. Following the preparation of the protein structure and the energy minimization of the fluorophores in PyRx, the docking simulations were completed. The resulting 2D and 3D interactions were then visualized utilizing Discovery Studio 2025 Visualizer [67] and ChimeraX 1.9 [68,69] softwares, respectively.

The docking study yielded the pose for every fluorophore inside the protein, each of which had its binding affinity score (Table 3). The docking study in BSA showed that the binding affinity scores for the fluorophores ranged from −7.40 to −7.70 kcal/mol. Fluorophore **6c** had the highest score of −7.70 kcal/mol, followed by **6b** with −7.60 kcal/mol, then **6a** with −7.40 kcal/mol. It was noticed that binding score increases with increasing alkyl chain length, owing to increasing the hydrophobic interactions.

**Figure 4:**
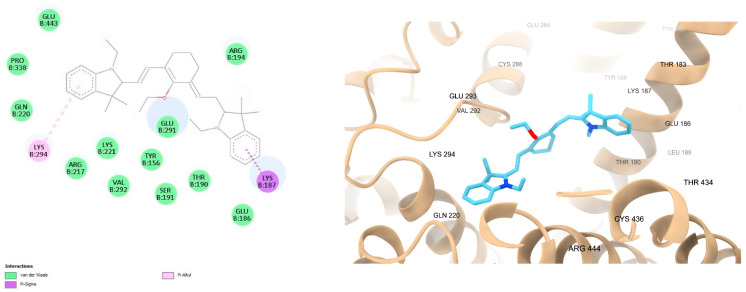
2D and 3D interaction diagram of fluorophore **6a** with bovine serum albumin (BSA, PDB ID: 4jk4); In the 3D diagram, **6a** shown as blue sticks (carbon atoms appear as blue, nitrogen atoms appear as dark blue, and oxygen atom appears as red), BSA shown as beige ribbons.

Studying the binding site in detail showed that the fluorophores tend to bind in domains II and III of the bovine serum albumin [70]. For **6a** (Figure 4 and Figure S19), the binding interactions included a pi–sigma interaction with lysine 187 (LYS187) and a pi–alkyl interaction with lysine 294 (LYS294), and the rest were just van der Waals interactions in the hydrophobic pockets of the binding site.

A pi–alkyl interaction occurs between the electron cloud of an aromatic ring (the pi system) and an alkyl group (a carbon-hydrogen chain). It is primarily a type of hydrophobic or van der Waals interaction. The electrons in the aromatic ring interact with the non-polar alkyl chain of an amino acid (here it is lysine 294). A pi–sigma interaction occurs between the pi electron cloud of an aromatic ring and a sigma bond (a single covalent bond) of another molecule (here it is lysine 187). Most commonly, this involves a polarized carbon–hydrogen (C-H) bond. The electron-rich pi cloud of the aromatic ring acts as a weak base (or electron donor), while the slightly positive hydrogen atom in a sigma bond acts as a weak acid (or electron acceptor). It is essentially a very specific, weak type of hydrogen bond (often called a C-H···π interaction).

**Figure 5:**
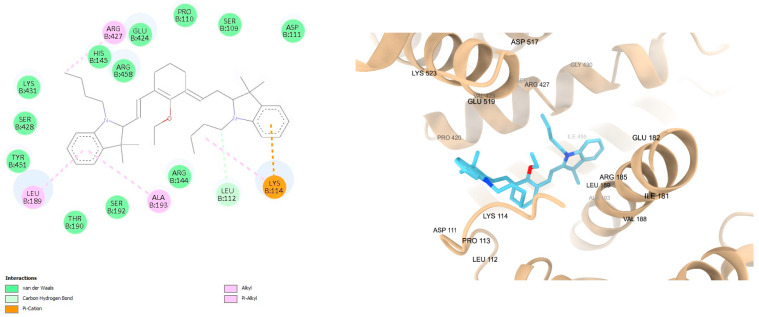
2D and 3D interaction diagram of fluorophore **6b** with bovine serum albumin (BSA, PDB ID: 4jk4); In the 3D diagram, **6b** shown as blue sticks (carbon atoms appear as blue, nitrogen atoms appear as dark blue, and oxygen atom appears as red), BSA shown as beige ribbons.

For fluorophore **6b** (Figure 5 and Figure S20), the interactions were mainly van der Waals interactions, in addition to pi–cation interaction with lysine 114 (LYS114), alkyl–hydrophobic interactions with arginine 427 (ARG427), and pi–alkyl interactions with leucine 189 (LEU189) and alanine 193 (ALA193). The increased number of interactions and their strength caused the binding affinity score to increase compared to fluorophore **6a**. A pi–cation interaction is a strong electrostatic attraction between a positively charged ion (a cation, in this case, the amino group of lysine) and the negatively charged electron cloud (the pi system) that sits above and below an aromatic ring. Aromatic rings have delocalized electrons that form a ring of negative charge. The amino group of lysine at physiological pH is protonated and has a full positive charge and comes near the face of that ring, and the opposite charges attract each other strongly.

**Figure 6:**
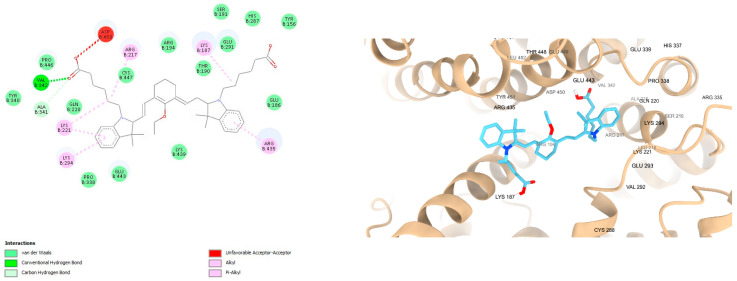
2D and 3D interaction diagram of fluorophore **6c** with bovine serum albumin (BSA, PDB ID: 4jk4); In the 3D diagram, **6c** shown as blue sticks (carbon atoms appear as blue, nitrogen atoms appear as dark blue, and oxygen atoms appear as red), BSA shown as beige ribbons.

Regarding fluorophore **6c** (Figure 6 and Figure S21), there were slightly less van der Waals interactions, due to the increased polarity by the carboxylic acid groups, but there were still pi–alkyl interactions with arginine 435 (ARG435), lysine 221 (LYS221), and lysine 294 (LYS294). The carboxylic acid group was involved in an extra hydrogen bond with valine 342 (VAL342), in addition to an unfavorable acceptor–acceptor interaction with aspartic acid 450 (ASP450).

### 2.7. Bovine Serum Albumin (BSA) Sensing

**Figure 7:**
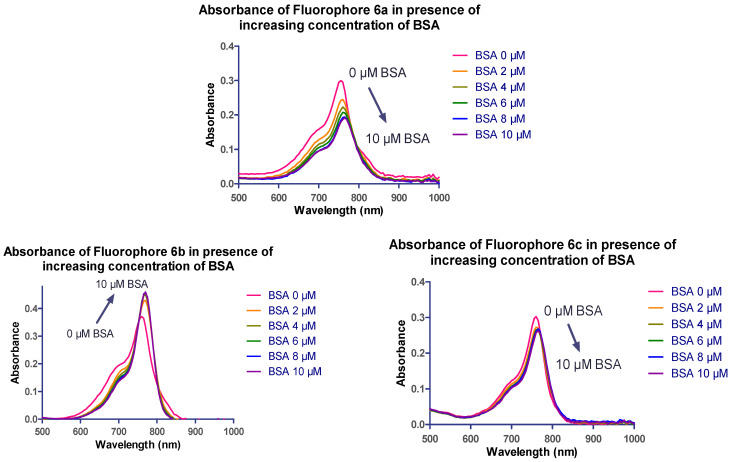
Absorbance spectra of fluorophores **6a** (4 μM), **6b** (4 μM), and fluorophore **6c** (8 μM) at increasing concentrations of bovine serum albumin (BSA) in 1X PBS.

After the docking study, the results were validated experimentally by examining the effect of adding BSA on the absorbance and fluorescence of fluorophores **6a–c**. The test was conducted by measuring the absorbance and fluorescence of fluorophores **6a–c** in the presence of increasing concentrations of BSA. For the absorbance, fluorophores **6a–c** were used in PBS buffer, and their absorbance was measured at different concentrations of BSA (Figure 7).

Fluorophore **6a** showed a slight bathochromic shift and hypochromic behavior with increasing concentration of BSA. Inversely for fluorophore **6b**, hyperchromic and bathochromic shifts were observed upon the addition of BSA, where the wavelength maxima shifted from 755 nm to 770 nm, and the absorbance jumped from 0.36 to 0.41. Upon increasing the BSA concentration, the absorbance kept increasing gradually until it reached 0.46. Fluorophore **6c** with the carboxylic acid groups had the smallest change in absorbance intensity with the addition of BSA. Although **6c** had the highest binding affinity score in the docking study, at the same time, it showed an unfavorable acceptor–acceptor interaction with one of the aspartic acid amino acids in the BSA chain. This unfavorable interaction could be the reason for the weaker interaction in the experimental results because of the increased percentage of aspartic acid and glutamic acid residues in the BSA chains [71,72], so this interaction could be a common interaction and prevent fluorophore **6c** from binding stably in the pockets of the BSA.

This different behavior could also be attributed to the difference in the hydrophobicity and log D between the various fluorophores, where BSA is known to bind more to hydrophobic small molecules. So, as the hydrophobicity decreased from **6b** (the one with the highest hydrophobicity and the highest predicted log D values) to **6c** (the one with the lowest hydrophobicity and the lowest predicted log D values), the level of binding and interaction decreased, which was manifested as a lower level of absorbance change.

**Figure 8:**
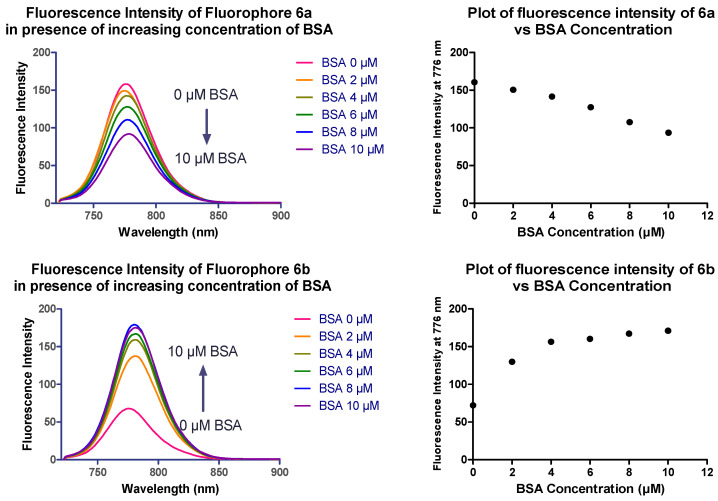

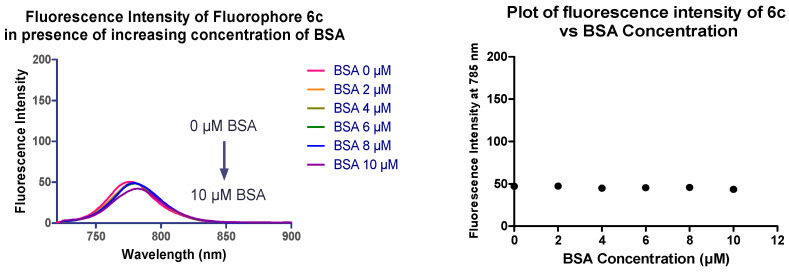
Fluorescence spectra of fluorophores **6a** (0.18 μM), **6b** (0.03 μM) and **6c** (0.18 μM) at increasing concentrations of bovine serum albumin (BSA) in 1X PBS, and plots of fluorescence intensity vs. BSA concentration for each fluorophore.

It was also important to test the sensing ability of the fluorophores using fluorescence titration to examine the effect of binding to BSA on the fluorescence intensity of the fluorophores. For this titration study, solutions of fluorophores **6a–c** were used in PBS buffer, and the fluorescence intensity was measured at different concentrations of BSA (Figure 8). Fluorophores **6a** and **6c** showed a similar behavior to their absorbance titration study, where the fluorescence intensity decreased upon increasing the concentration of BSA. Fluorophore **6a** had a small change in fluorescence, where the intensity changed from 161 at 0 μM of BSA to 89 at 10 μM of BSA.

Interestingly, for fluorophore **6b** a similar observation happened as the absorbance, where the emission wavelength maximum red-shifted from 768 nm to 782 nm, and fluorescence enhancement was observed as a jump in the fluorescence intensity from 65 at 0 μM BSA to 136 at 1 μM BSA. The fluorescence intensity increased upon the addition of higher concentrations of BSA until it reached 177 at 10 μM of BSA, which is considered 3× enhancement upon addition of BSA.

Fluorophore **6c** was again the weakest in interaction and the smallest in the observable change in fluorescence intensity, owing to its acidic carboxylic groups. The change in behavior with BSA of fluorophores **6a** and **6c** compared to fluorophore **6b** was worth noting and required more mechanistic studies to understand the nature and mechanism of binding, which will be studied in detail in the future studies of this project.

The contrasting optical responses of fluorophores **6a–c** toward BSA can be rationalized by two competing photophysical pathways that operate upon protein binding [27,28,30]. The first is a fluorescence-enhancement pathway: in aqueous buffer these heptamethine cyanines undergo extensive non-radiative decay through excited-state torsional motion of the polymethine chain [18] and through the formation of non-emissive H-aggregates, which accounts for their low quantum yields in HEPES and PBS buffers (Table 1) [28,30,41,42]. Insertion of the dye into a hydrophobic albumin pocket simultaneously disaggregates the chromophore into emissive monomers, rigidifies it so that torsional non-radiative decay is suppressed, and shields it from water, all of which raises the quantum yield and give a hyperchromic, red-shifted turn-on response [31,32].

The second is an opposing quenching pathway, in which proximity of the bound dye to the electron-rich aromatic residues of BSA (tryptophan and tyrosine) enables photoinduced electron transfer to the electron-poor cationic chromophore, while the formation of a non-fluorescent ground-state dye–protein complex contributes static quenching [73,74,75]. The net response of each fluorophore therefore reflects the balance between these two pathways, which is in turn governed by the dye’s hydrophobicity and the depth of its insertion into the protein. For the most hydrophobic dye **6b** (log D 7.00), which is also the least emissive in buffer and engages the most extensive set of hydrophobic contacts in the docking study, deep and complete burial within the binding pocket makes the enhancement pathway dominant, producing the observed rise in both absorbance and fluorescence.

In contrast, fluorophore **6a** (log D 4.55) is less hydrophobic and remains substantially monomeric and emissive in buffer, so little additional enhancement is available upon binding; its shallower, more solvent-exposed association allows the quenching pathway to prevail, and the accompanying hypochromic change in absorbance is itself consistent with ground-state complex formation. Fluorophore **6c** (log D 3.12) represents the limiting case, as at physiological pH its carboxypentyl groups are deprotonated, rendering the dye anionic and the least hydrophobic of the series; the resulting electrostatic repulsion from the net-negatively charged BSA surface, together with the unfavorable acceptor–acceptor contact with Asp450 identified in the docking study, prevents stable insertion and yields the smallest spectral change [70,71]. Importantly, the bathochromic shift common to all three dyes confirms that each associate with the less polar protein microenvironment [55]; only the sign of the intensity change differs, as set by the enhancement–quenching balance.

The BSA binding behavior of fluorophores **6a–c** is consistent with previously reported data for structurally related polymethine–albumin systems. The ~15 nm bathochromic shift in both absorption (755→770 nm) and emission (768→782 nm), observed for **6b** upon BSA binding falls within the 13–23 nm range reported for bridged pentamethine cyanine dyes on serum-albumin association [76], and the accompanying hyperchromic, turn-on response mirrors the enhancement of monomer absorbance and fluorescence quantum yield documented for cyanine dyes binding HSA and BSA [31,32,77]. The underlying mechanism is also shared: heptamethine cyanines bearing a chloro-substituted cyclohexenyl bridge, structurally analogous to the parent dyes **5a–c**, are known to form non-emissive H-type aggregates in aqueous buffer and to disaggregate into emissive monomeric species upon albumin binding [19], in line with the disaggregation-driven turn-on proposed here.

Our own previously reported peptidomimetic heptamethine cyanines behaved analogously, being essentially non-fluorescent in aqueous media and switching on upon protein binding [34]. The computed binding affinities (−7.4 to −7.7 kcal/mol) lie within the −6 to −10 kcal/mol range typically obtained for dye–albumin complexes using comparable docking protocols [78], and the predicted association with domains II and III is consistent with the principal ligand-binding regions of albumin (Sudlow sites I and II, located in subdomains IIA and IIIA) [79].

**Figure 9:**
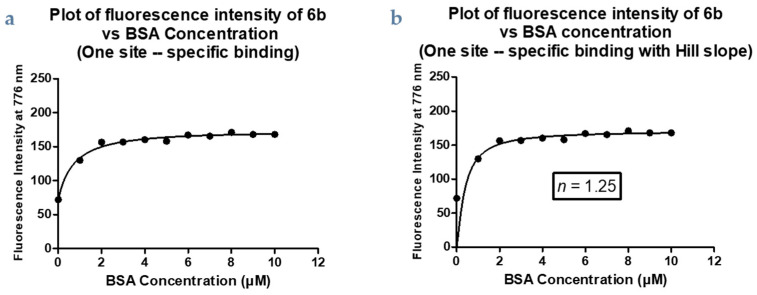
Non-linear curve fitting for fluorescence intensity of **6b** (0.03 μM) vs. BSA concentration; (**a**): using one-site-specific binding curve, (**b**): using one-site-specific binding curve with Hill slope.

The binding dissociation constant (K_d_) was calculated for fluorophore **6b** (the strongest binder) to BSA by plotting the fluorescence intensity at its maximum fluorescence intensity wavelength vs. BSA concentration using a one-site binding non-linear regression curve (Figure 9a). The points have a good fit using this graph, with an R^2^ value of 0.9868 confirming the excellent fit to this equation and indicating a 1:1 binding stoichiometry. K_d_ was calculated to be 0.7469 µM, and K_a_ was calculated to be 1.3389 µM^−1^, showing the high affinity between fluorophore **6b** and BSA. Using the linear range of the fluorescence intensity vs. BSA concentration plot (Figure S22), the limit of detection (LOD) and limit of quantitation (LOQ) were calculated. LOD was calculated to be 1.9 µM, and LOQ was calculated to be 6.0 µM, indicating the high sensitivity of fluorophore **6b** to BSA.

To verify the binding stoichiometry of fluorophore **6b** with BSA, two methods were used. Firstly, from the plot of fluorescence intensity of **6b** vs. BSA concentration (Figure 9a), the curve appears hyperbolic without a sigmoidal shape, which is consistent with *n* = 1 (1:1 binding). The single K_d_ value also supports this. This was verified by plotting the data as a one-site-specific binding with Hill slope (Figure 9b), and the binding stoichiometry was determined to be *n* = 1.25, suggesting a 1:1 interaction between compound **6b** and BSA.

**Figure 10:**
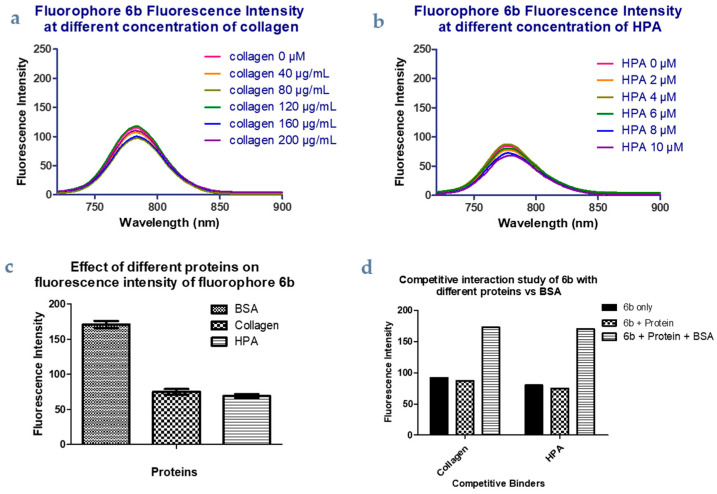
Selectivity studies for fluorophore **6b** (0.03 μM); (**a**): fluorescence intensity with collagen titration, (**b**): fluorescence intensity with HPA titration, (**c**): bar graph showing the effect of different proteins (collagen or HPA or in the presence of BSA) on fluorescence intensity of **6b**, (**d**): competitive binding study.

The fluorescence spectrum of fluorophore **6b** was recorded with the titration of different proteins as collagen and human parvalbumin to check whether it would show the same change in fluorescence intensity or not (Figure 10a,b). Neither of these proteins caused the fluorescence enhancement of fluorophore **6b**, which showed that these fluorophores have selectivity towards BSA (Figure 10c). Competitive study of the fluorophore with either of the non-interacting proteins upon addition of BSA also showed fluorescence enhancement of **6b**, indicating the strong binding and affinity of fluorophore **6b** towards BSA (Figure 10d).

**Figure 11:**
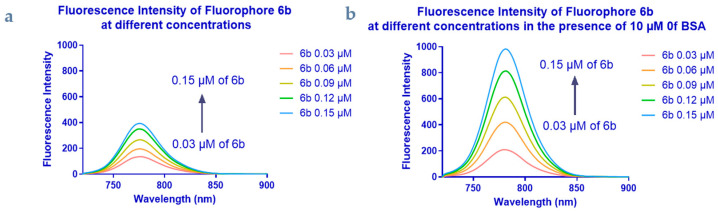
Fluorescence titration of fluorophore **6b** (0.03–0.15 μM); (**a**): fluorescence titration in absence of BSA, (**b**): fluorescence titration in presence of 10 μM BSA.

The effect of changing the dye concentration on the fluorescence intensity was studied both in the absence and in the presence of a fixed concentration of BSA (Figure 11a,b). This study was done by measuring the fluorescence intensity of two sets of mixtures with an increasing concentration of fluorophore **6b**; one set does not have BSA in solution, and the other has 10 μM BSA. Both sets showed an increase in fluorescence by increasing the concentration of the fluorophore; however, the set that has BSA in solution showed a much higher fluorescence intensity for each corresponding concentration, indicating the fluorescence enhancement and the breaking of the aggregation that is formed in the absence of BSA.

## 3. Conclusions

The work presented here showed that substituting the meso-chlorine atom of a heptamethine carbocyanine dye with an ethoxy group leads to a noticeably more stable cyanine without disrupting its basic NIR character. Fluorophores **6a–c** handled prolonged irradiation better than ICG, where they showed stronger photothermal stability upon continuous irradiation and preserved their absorbance. DFT studies were conducted to predict their HOMO-LUMO gap, giving an indication and confirming their experimental absorbance wavelengths. In addition, owing to their slightly hydrophobic nature, they were tested for interactions with bovine serum albumin both in silico and in cuvette.

Fluorophores **6a–c** showed interesting behavior when exposed to bovine serum albumin. Fluorophore **6a** showed an absorbance decrease and fluorescence quenching upon the addition of BSA. Fluorophore **6c** was the weakest in the effect of BSA addition, mostly due to its carboxylic acid groups and slightly hydrophilic nature. Notably, fluorophore **6b** showed a strong response in absorbance and fluorescence enhancement when exposed to BSA, which indicated that the dye was readily accommodated within the protein’s hydrophobic regions. This confirmed the docking study results that showed the hydrophobic interactions between the fluorophore and the protein’s hydrophobic pocket amino acids. Although its fluorescence in aqueous media remained modest because of its hydrophobic nature, the sharp increase in signal upon BSA binding demonstrated that the substitution did not impair the dye’s ability to act as a probe. Non-linear regression analysis of the fluorescence titration data yielded a dissociation constant (K_d_) of 0.75 μM and a Hill coefficient of *n* = 1.25, consistent with approximately 1:1 stoichiometric binding. The LOD and LOQ for BSA detection were calculated to be 1.9 and 6.0 μM, respectively, reflecting the high sensitivity of the probe. Furthermore, selectivity studies confirmed that fluorophore **6b** does not produce fluorescence enhancement with other proteins such as collagen or human parvalbumin, underscoring its selectivity toward BSA. Collectively, these results establish meso-alkoxy substitution as a promising design strategy for developing more durable, selective NIR probes, and future work will explore similar modifications in related cyanine scaffolds.

## 4. Experimental

### 4.1. Materials and Methods

Chemicals used in the synthesis are American Chemical Society or HPLC grade, purchased from Sigma Aldrich (Saint Louis, MO, USA), Thermo Fisher Scientific and TCI America (Waltham, MA, USA). The ^1^HNMR (400 MHz), ^13^CNMR (101 MHz), and COSY NMR (400 MHz) spectra were recorded using a Bruker Avance 400 MHZ spectrometer (Bruker Corporation, Billerica, MA, USA) with CDCl_3_ (Sigma-Aldrich, Burlington, MA, USA) and MeOD (Sigma-Aldrich, Burlington, MA, USA), containing tetramethylsilane (TMS) as an internal calibration standard. Chemical shifts were reported in parts per million (ppm). The following abbreviations are used for signal multiplicity: s (singlet), d (doublet), t (triplet), q (quartet), p (pentet), m (multiplet), and br (broad). Coupling constants (J) are provided in hertz (Hz). The melting points (mp) were measured with open Pyrex capillary tubes (Corning, Corning, NY, USA) and Thomas Hoover apparatus (Thomas Hoover & Company, Sheffield Village, OH, USA). The absorbance and fluorescence properties were measured using a Varian Cary 50 spectrophotometer (Varian Inc., Santa Clara, CA, USA) and Shimadzu RF-5301 PC spectrofluorometer (Shimadzu Scientific Instruments, Columbia, MD, USA), respectively. The VWR disposable two-sided polystyrene cuvettes with path length 1 cm were utilized to dissolve the dye in solvents for measurement. The quantum yields of dyes were measured according to the reported method with reference to indocyanine green (ICG) [80].

### 4.2. Chemistry–Experimental

#### 4.2.1. Synthetic Procedure for the Preparation of Indolium Salt (**2A–C**)

The indolium salts **2a–c** were prepared using an established in-house method [2,33]. The substituted indole **1** (3.0 g, 19 mmol, 1 eq) was reacted with various alkyl halides (3 eq) in acetonitrile (10 mL) at 90 °C for 48 h. The reaction’s progress was monitored by TLC. After completion, the mixture was concentrated under vacuum, and the crude product was crystallized from methanol/diethyl ether or methanol/ethyl acetate to afford the final products of **2a–c** as powders with different shades of red (56–98%).

#### 4.2.2. Synthesis of the Vilsmeier–Haack Linker for the Heptamethine Cyanine Dye (**4**)

The linker for the heptamethine cyanine dye was obtained through the Vilsmeier–Haack Chloroformylation as reported earlier [3,81,82]. Dimethyl formamide (DMF) (13 mL, 163 mmol, 3.2 eq) was dissolved in dichloromethane (13 mL) and cooled to 0 °C in an ice bath. Phosphorus oxychloride POCl_3_ (11 mL, 115 mmol, 2.3 eq) dissolved in dichloromethane was added dropwise at 0 °C to the DMF solution and stirred for 30 min. Cyclohexanone **5** (5.3 mL, 51 mmol, 1 eq) was added while in the ice bath, then the reaction mixture was heated at 70 °C for 4 h. The reaction was monitored with TLC, and after its completion, it was quenched by pouring the reaction mixture on ice/water mixture (1000 mL) and stirring at room temperature overnight. The formed precipitate was filtered and washed with distilled water to obtain the product of **6** as a yellow solid (5.6 g, 70%).

#### 4.2.3. Synthetic Procedure for the Preparation of Heptamethine Cyanine Dye (**5A–C**)

The heptamethine carbocyanine dyes **5a–c** were synthesized as reported earlier by our group [3,7,34,82]. The Vilsmeier linker **4** (1 eq) was mixed in acetic anhydride (7 mL), with the indolium salts **2a–c** (1.0 g, 2 eq) in the presence of sodium acetate (2 eq) as a base. The reaction mixture of each reaction was heated in an oil bath to 70 °C for 4 h (**5a** and **5b**) or kept at room temp. for 1 h (**5c**). Thin Layer Chromatography (TLC) and UV-vis spectroscopy were used to follow up the reaction completion, then diethyl ether (100 mL) was added to precipitate the product, which was filtered. Then, the crude product was purified by recrystallization by dissolving in methanol (1.5 mL) and precipitating in ethyl acetate (100 mL) to obtain the heptamethine dye **5a–c** as green solids (61–74%).

#### 4.2.4. Synthetic Procedure for the Heptamethine Carbocyanine Meso-Modified Fluorophores (**6A–C**)

Potassium carbonate (1 eq) was mixed with ethanol, which was used as a solvent and a reagent (8 mL, excess), and stirred at room temperature for 30 min. The heptamethine cyanine dyes **5a–c** (400 mg, 1 eq) were added, and the reaction mixture was refluxed at 85 °C for 8 h. TLC and UV-vis spectroscopy were used to monitor the reaction by observing the change in the wavelength of the absorption bands from 775 nm for the heptamethine monomer dye to 755 or 760 nm for the meso-modified dyes. The reaction mixture was concentrated in vacuo, and the residue was purified by neutral alumina or silica gel flash column chromatography using DCM/MeOH, then recrystallization by dissolving in methanol (1.0 mL) and precipitating in diethyl ether (100 mL) to obtain the final dyes of **6a–c** as green solids. The synthesized dye was characterized by ^1^HNMR, ^13^CNMR, HRMS and COSY NMR and was determined to be >95% pure.

*2-((E)-2-((E)-2-ethoxy-3-(2-((E)-1-ethyl-3,3-dimethylindolin-2-ylidene)ethylidene)cyclohex-1-en-1-yl)vinyl)-1-ethyl-3,3-dimethyl-3H-indol-1-ium iodide* **6a**. Isolated by neutral alumina column chromatography using DCM/MeOH 98:2 as a green solid. Yield (137 mg, 34%); mp 155–157 °C; ^1^H NMR (400 MHz, CDCl_3_) δ 8.11 (d, *J* = 14.1 Hz, 2H), 7.38 (t, *J* = 7.4 Hz, 4H), 7.22 ((t, *J* = 7.4 Hz, 2H), 7.17 (d, *J* = 7.4 Hz, 2H), 6.06 (d, *J* = 14.1 Hz, 2H), 4.19 (q, *J* = 7.3 Hz, 4H), 4.12 (q, *J* = 7.1 Hz, 2H), 2.62 (br t, 4H), 1.94 (br p, 2H), 1.71 (s, 12H), 1.60 (t, *J* = 7.1 Hz, 3H), 1.46 (t, *J* = 7.3 Hz, 6H); ^13^C NMR (101 MHz, CDCl_3_) δ 172.0, 171.3, 142.4, 141.7, 141.2, 129.2, 125.3, 124.0, 122.6, 110.8, 99.5, 73.9, 49.4, 40.1, 28.7, 25.1, 21.5, 16.6, 12.7; HRMS (ESI) Calcd. for [C_36_H_45_N_2_O]^+^ *m*/*z* 521.3532, found *m*/*z* 521.3517; λ_abs_ = 755 nm in EtOH.

*1-butyl-2-((E)-2-((E)-3-(2-((E)-1-butyl-3,3-dimethylindolin-2-ylidene)ethylidene)-2-ethoxycyclohex-1-en-1-yl)vinyl)-3,3-dimethyl-3H-indol-1-ium iodide* **6a**. Isolated by neutral alumina column chromatography using DCM/MeOH 99:1 as a green solid. Yield (145 mg, 36%); mp 160–162 °C; ^1^HNMR (400 MHz, CDCl_3_) δ 8.05 (d, *J* = 14.0 Hz, 2H), 7.32 (t, *J* = 7.6 Hz, 4H), 7.16 (t, *J* = 7.6 Hz, 2H), 7.07 (d, *J* = 7.6 Hz, 2H), 5.99 (d, *J* = 14.0 Hz, 2H), 4.08 (br t, 4H), 4.03 (br q, 2H), 2.56 (t, *J* = 6.4 Hz, 4H), 1.90–1.87 (m, 2H), 1.77 (p, *J* = 7.7 Hz, 4H), 1.66 (s, 12H), 1.54 (t, *J* = 7.1 Hz, 3H), 1.48–1.40 (m, 4H), 0.97 (t, *J* = 7.4 Hz, 6H); ^13^CNMR (101 MHz, CDCl_3_) δ 171.9, 171.7, 142.8, 141.5, 141.1, 129.1, 125.2, 123.9, 122.6, 110.9, 99.8, 73.9, 49.3, 44.8, 29.7, 28.7, 25.0, 21.5, 20.8, 16.6, 14.3; HRMS (ESI) Calcd. for [C_40_H_53_N_2_O]^+^ *m*/*z* 577.4158, found *m*/*z* 577.4180; λ_abs_ = 760 nm in EtOH.

*1-(5-carboxypentyl)-2-((E)-2-((E)-3-(2-((E)-1-(5-carboxypentyl)-3,3-dimethylindolin-2-ylidene)ethylidene)-2-ethoxycyclohex-1-en-1-yl)vinyl)-3,3-dimethyl-3H-indol-1-ium bromide* **6c**. Isolated by silica gel column chromatography using DCM/MeOH 95:5 as a green solid. Yield (100 mg, 25%); mp 166–168 °C; ^1^H NMR (400 MHz, MeOD) δ 8.22 (d, *J* = 14.2 Hz, 2H), 7.52 (d, *J* = 7.7 Hz, 2H), 7.44 (t, *J* = 7.7 Hz, 2H), 7.29 (d, *J* = 7.7 Hz, 4H), 6.17 (d, *J* = 14.2 Hz, 2H), 4.18 (t, *J* = 7.4 Hz, 4H), 4.13 (br q, 2H), 2.67 (t, *J* = 6.2 Hz, 4H), 2.26 (t, *J* = 7.4 Hz, 4H), 1.97 (p, *J* = 6.2 Hz, 2H), 1.90 (p, *J* = 7.4 Hz, 4H), 1.76 (s, 12H), 1.72 (br p, 4H), 1.64 (t, *J* = 7.0 Hz, 3H), 1.53 (p, *J* = 7.4 Hz, 4H), ^13^C NMR (101 MHz, MeOD) δ 183.1, 173.3, 172.7, 143.9, 142.5, 142.4, 129.9, 126.1, 124.5, 123.6, 111.9, 100.6, 74.7, 50.3, 45.0, 38.5, 28.7, 28.2, 27.9, 27.1, 25.7, 22.6, 16.6; HRMS (ESI) Calcd. for [C_44_H_57_N_2_O_5_]^+^ *m*/*z* 693.4267, found *m*/*z* 693.4290; λ_abs_ = 760 nm in EtOH.

### 4.3. Photophysical Properties Studies

The optical characteristics of the synthesized fluorophores were examined after preparing a 1 mM stock solution in DMSO. Spectral data (absorbance and fluorescence emission) were collected in four media: ethanol, DMSO, HEPES buffer, and PBS. A Varian Cary 50 spectrophotometer was used for absorbance measurements, and a Shimadzu RF-5301PC spectrofluorometer (Shimadzu Corporation, Kyoto, Japan) was used for fluorescence emission studies.

### 4.4. Quantum Yield of Fluorescence (ΦF) Calculation

The quantum yield of fluorescence was measured in ethanol, DMSO, HEPES buffer, and PBS buffer using a Shimadzu RF-5301PC spectrofluorometer. The calculation involved comparing the dye’s fluorescence intensity to that of the ICG standard, which was selected due to its structural similarity (heptamethine cyanine dye). Literature values of ICG quantum yield (14% in ethanol [10], 16.7% in DMSO [9], and 2.9% in water-based buffers [10]) were used for the comparison. The measurements were conducted using a 700 nm excitation wavelength, 5 nm slit widths, and dye concentrations ranging from 0.018 to 0.36 µM.

### 4.5. Photothermal Stability Studies

To evaluate the fluorophores photostability, solutions of the fluorophores in ethanol (4 μM) were divided into two sets: a dark control (stored at 25 °C and covered) and an irradiated set. The irradiated set was continuously exposed to a 6000 mW 254 nm UV lamp light placed 10 cm away from the fluorophores’ solutions at 25 °C. This second set was covered to prevent any other light from interfering. The absorbance of samples from both sets was monitored and measured at 24 h intervals over a period of 72 h, and the samples were returned to the vials and stored for the next round of measurements.

### 4.6. Density Functional Theory (DFT) Study

The density functional theory (DFT) calculations were performed in Spartan^®^ 24 software [61]. Each molecule was first imported as a *.mol* file and automatically converted from its 2D drawing into a 3D structure. An initial geometry optimization was carried out in the gas phase using the “Equilibrium Geometry” routine with the MMFF molecular mechanics force field. Following this pre-optimization step, a full DFT geometry optimization was conducted in the gas phase using the “Equilibrium Geometry” method with the B3LYP functional and the 6-31G* polarized basis set. The resulting frontier molecular orbital energies were then obtained and expressed in electron volts (eV).

### 4.7. Molecular Docking Study

The energy of the fluorophore ligands was minimized using Spartan 24^®^ software [61], and the resulting structures were imported into PyRx 0.8 as .mol files. The crystal structure of bovine serum albumin (BSA; PDB ID: 4JK4) was retrieved from the Research Collaboratory for Structural Bioinformatics (RCSB) Protein Data Bank to serve as the receptor. For bovine serum albumin, the crystal structure with PDB ID 4JK4 was used in the docking study [67]. To prepare the protein, identical chains were removed using Discovery Studio 2025 Visualizer [68], and the refined structure was then saved as a PDB file and designated as a macromolecule within the PyRx 0.8 software [66] using the “Make macromolecule” command. Docking simulations were performed using AutoDock Vina 1.1.2, integrated within PyRx 0.8, after the ligands were converted to the required pdbqt format using “Convert to Autodock Ligand (pdbqt)” command. Because the specific binding sites on the BSA protein were not predetermined, a blind docking approach was employed. To capture any potential interactions, the grid box was established to encompass the entire protein chain, utilizing the dimensions of X: 91.3471, Y: 61.5461, and Z: 73.2728 Å.

Upon completion of the simulations, the resulting docking poses were exported as PDB files, with their corresponding binding affinities recorded in kcal/mol. To analyze the receptor–ligand relationships, these poses were imported into Discovery Studio 2025 Visualizer, where 2D interaction diagrams were generated. The protein structure was marked as the receptor, and the fluorophore pose was marked as the ligand; then, the “Show 2D diagram” command was chosen to show the 2D interaction diagrams. Finally, 3D representations of the binding environment were captured by mapping the poses in ChimeraX 1.9, with the protein receptor rendered in both standard cartoon and molecular surface formats [69,70].

## Figures and Tables

**Table 1:** Optical properties of fluorophore **6a–c** and ICG in different solvents.

Fluorophore	Solvent	Absorbance Wavelength Maxima (nm)	Excitation Wavelength (nm)	Emission Wavelength (nm)	Stokes Shift (nm)	Extinction Coefficient (M^−1^cm^−1^)	Quantum Yield of Fluorescence (%)	Molecular Brightness (M^−1^cm^−1^)
**Fluorophore 6a**	Ethanol	755	700	770	15	150,600	9.27	13,967
	DMSO	770	700	786	16	112,300	9.99	11,224
	HEPES Buffer ^a^	755	700	772	17	61,800	0.357	220.4
	PBS ^b^	755	700	768	13	47,900	2.23	1068
**Fluorophore 6b**	Ethanol	760	700	774	14	216,400	17.3	37,437
	DMSO	770	700	789	19	168,600	12.0	20,232
	HEPES Buffer ^a^	755	700	776	21	176,200	0.418	736.5
	PBS ^b^	760	700	778	18	120,000	0.946	1135
**Fluorophore 6c**	Ethanol	760	700	777	17	63,000	5.43	3427
	DMSO	770	700	791	21	52,100	13.74	7158
	HEPES Buffer ^a^	760	700	776	16	27,400	2.39	656.7
	PBS ^b^	760	700	774	14	39,800	2.18	869.5
**ICG**	Ethanol	785	710	825	40	215,000	14.0 [10]	30,100
	DMSO	798	720	817	19	216,000	16.7 [9]	36,072
	HEPES Buffer ^a^	778	720	802	24	148,000	2.9 [10]	4292
	PBS ^b^	778	720	802	24	146,000	2.9 [10]	4234

^a^ HEPES buffer was used in a concentration of 50 mM with a pH of 7.4. ^b^ PBS was used in a concentration of 1X PBS, with a pH of 7.4.

**Table 2:** Physicochemical properties of fluorophores **6a–c** as predicted by Chemaxon^®^ Marvinsketch.

Fluorophore	MW (g/mol)	Log D	Rotatable Bonds	Surface Area (Å^2^)	TPSA (Å^2^)	Volume (Å^3^)	HBD/ HBA	Polarizability
**6a**	648.67	4.55	7	881.6	15.48	525.1	0/2	63.88
**6b**	704.78	7.00	11	1003	15.48	591.9	0/2	71.27
**6c**	773.85	3.12	17	1155	90.08	683.2	2/6	79.53

**Table 3:** Binding affinity scores of the synthesized fluorophores **6a–c** as predicted by PyRx software.

Fluorophore	Binding Affinity Score in BSA (kcal/mol)
**6a**	−7.4
**6b**	−7.6
**6c**	−7.7

## Data Availability

The original contributions presented in this study are included in the article/Supplementary Material. Further inquiries can be directed to the corresponding author.

## Supplementary Materials

The following supporting information can be downloaded at: https://www.mdpi.com/article/10.3390/molecules31132267/s1, Figure S1: ^1^H NMR spectrum of fluorophore 6a in CDCl_3_ (400 MHz), Figure S2: ^13^C NMR spectrum of fluorophore 6a in CDCl_3_ (101 MHz), Figure S3: HRMS of fluorophore 6a, Figure S4: COSY NMR spectrum of fluorophore 6a in CDCl_3_ (400 MHz), Figure S5: ^1^H NMR spectrum of fluorophore 6b in CDCl_3_ (400 MHz), Figure S6: ^13^C NMR spectrum of fluorophore 6b in CDCl_3_ (101 MHz), Figure S7: HRMS of fluorophore 6b, Figure S8: COSY NMR spectrum of fluorophore 6b in CDCl_3_ (400 MHz), Figure S9: ^1^H NMR spectrum of fluorophore 6c in CDCl_3_ (400 MHz), Figure S10: ^13^C NMR spectrum of fluorophore 6c in CDCl_3_ (101 MHz), Figure S11: HRMS of fluorophore 6c, Figure S12: COSY NMR spectrum of fluorophore 6c in CDCl_3_ (400 MHz), Figure S13: Structure of Indocyanine Green (ICG), Figure S14: Fluorophore 6a absorbance curves at different concentrations, calibration curves, and fluorescence curves (at concentrations of 0.072 to 0.36 μM) in different solvents, Figure S15: Fluorophore 6b absorbance curves at different concentrations, calibration curves, and fluorescence curves (at concentrations of 0.036 to 0.72 μM) in different solvents, Figure S16: Fluorophore 6c absorbance curves at different concentrations, calibration curves, and fluorescence curves (at concentrations of 0.36 μM) in different solvents, Figure S17: Frontier Molecular Orbitals (HOMO and LUMO) of fluorophore 6b as predicted by DFT studies, with an energy gap of 2.11 eV, Figure S18: Frontier Molecular Orbitals (HOMO and LUMO) of fluorophore 6c as predicted by DFT studies, with an energy gap of 2.09 eV, Figure S19: Fluorophore 6a pose inside bovine serum albumin (BSA, PDB ID: 4jk4), Figure S20: Fluorophore 6b pose inside bovine serum albumin (BSA, PDB ID: 4jk4), Figure S21: Fluorophore 6c pose inside bovine serum albumin (BSA, PDB ID: 4jk4), Figure S22: Linear range of fluorescence intensity of fluorophore 6b vs. BSA Concentration for calculation of LOD and LOQ, Video S1: Fluorophore 6a binding to BSA, Video S2: Fluorophore 6b binding to BSA, Video S3: Fluorophore 6c binding to BSA.
