# Supplementary material for: Ethoxy Meso-Modified Heptamethine Cyanine Fluorophores: Synthesis, Photophysical Properties and BSA Sensing Study
Source: Molecules. 2026 Jun 29;31(13):2267. doi: 10.3390/molecules31132267 (PMC13362738; doi:10.3390/molecules31132267)
Supplement: Supplementary file 1 [file molecules-31-02267-s001.zip › molecules-4334825-supplementary.pdf]

Supporting Information

Ethoxy Meso-Modified Heptamethine Cyanine Fluorophores: Synthesis, Photophysical Properties and BSA Sensing Studies

Tarek Erfan Ahmed[1], Maged Henary*[1,2]
[1]Department of Chemistry, Georgia State University, Atlanta, Georgia, 30303
[2]Center For Diagnostics and Therapeutics, Georgia State University, Atlanta, Georgia, 30303

*Address correspondence to
Dr. Maged Henary
Professor and Associate Chair of Chemistry
Department of Chemistry
Georgia State University
Atlanta, Georgia 30303
USA
mhenary1@gsu.edu
Phone: 404-413-5566

Table of Contents

1. ^{1}HNMR, ^{13}CNMR, COSY and HRMS Spectra of fluorophores 6a-c 3

2. Absorbance and Emission Spectra of fluorophores 6a-c 15

3. HOMO and LUMO orbitals of the fluorophores 18

4. Molecular Docking Study 20

Docking figures of the synthesized fluorophores with bovine serum albumin (BSA) 20

5. Limit of Detection (LOD) and Limit of Quantitation (LOQ) Calculation............................ 21

1. ^{1}HNMR, ^{13}CNMR, COSY and HRMS Spectra of fluorophores 6a-c

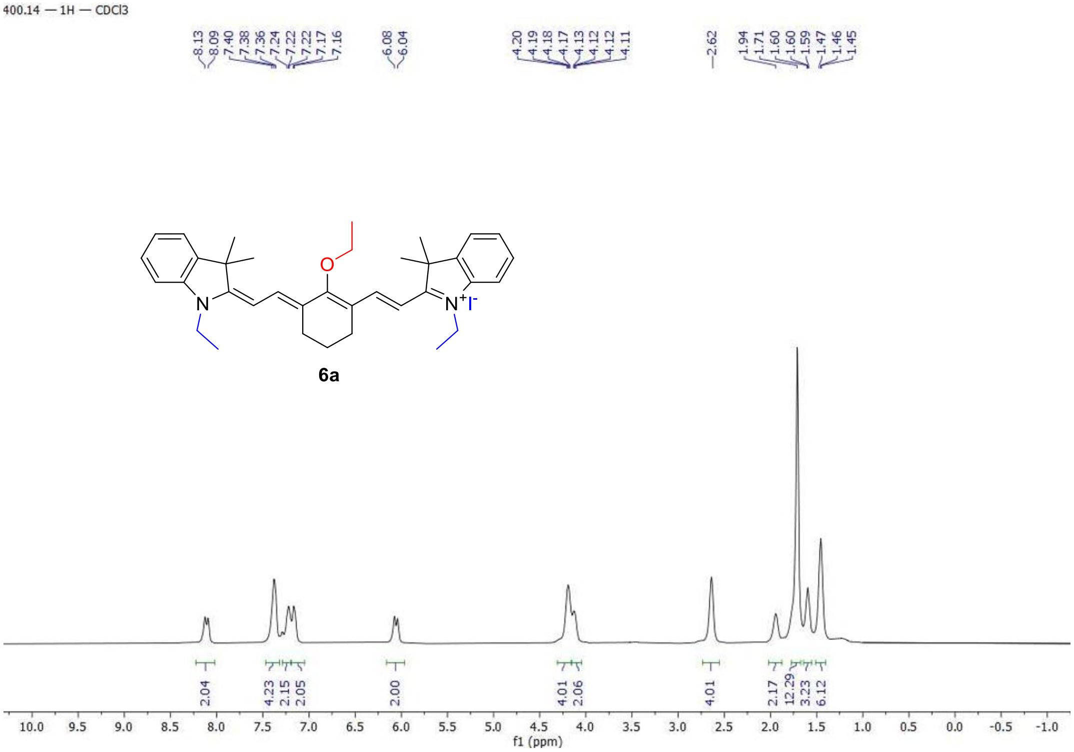

Figure S1. ^{1}HNMR spectrum of fluorophore **6a** in $CDCl_3$ (400 MHz)

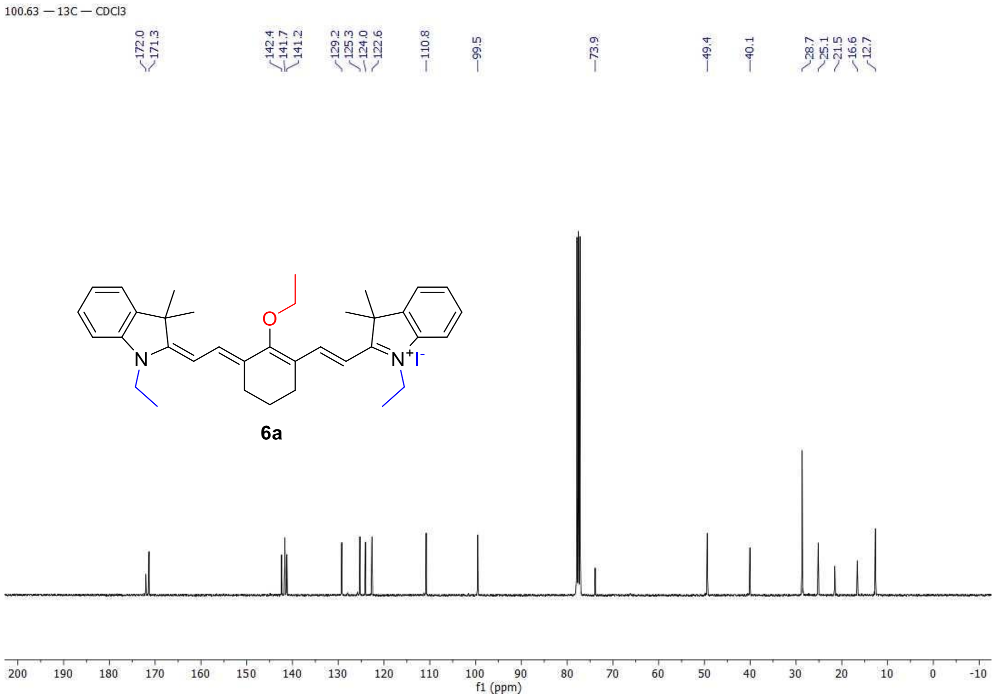

Figure S2. ^{13}CNMR spectrum of fluorophore **6a** in $CDCl_3$ (101 MHz)

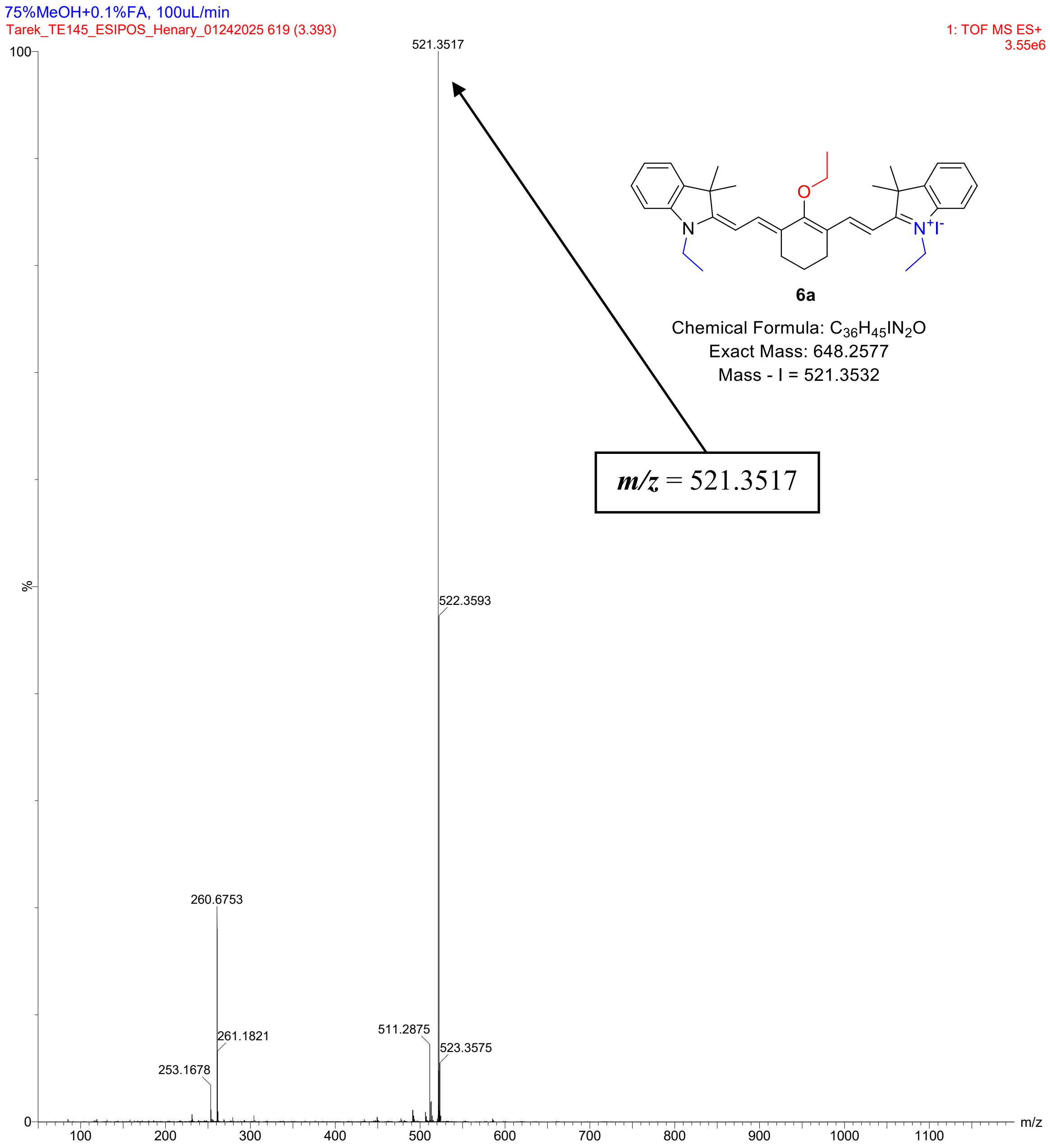

Figure S3. HRMS of fluorophore **6a**

Elemental Composition Report

Single Mass Analysis

Tolerance = 20.0 PPM / DBE: min = -50.0, max = 500.0

Element prediction: Off

Monoisotopic Mass, Even Electron Ions

5000 formula(e) evaluated with 1 result within limits (all results (up to 1000) for each mass)

Elements Used:

C: 36-36 H: 0-120 N: 0-20 O: 0-50 Na: 0-2 S: 0-3

Minimum:				-50.0		
Maximum:		1000.0		20.0	500.0	
Mass	Calc. Mass	mDa	PPM		DBE	Formula
521.3517	521.3532	-1.5	-2.9		15.5	C36 H45 N2 O

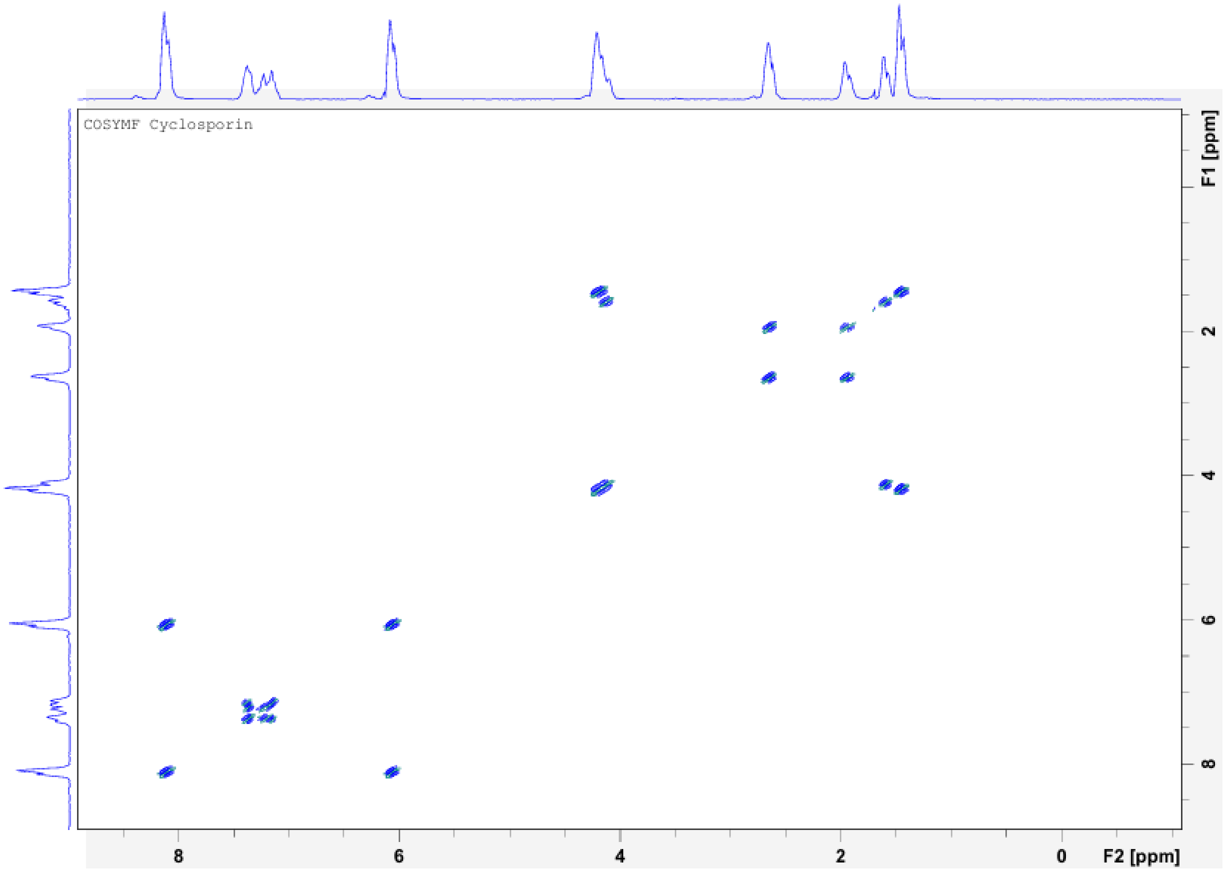

Figure S4. COSY NMR spectrum of fluorophore **6a** in $CDCl_3$ (400 MHz)

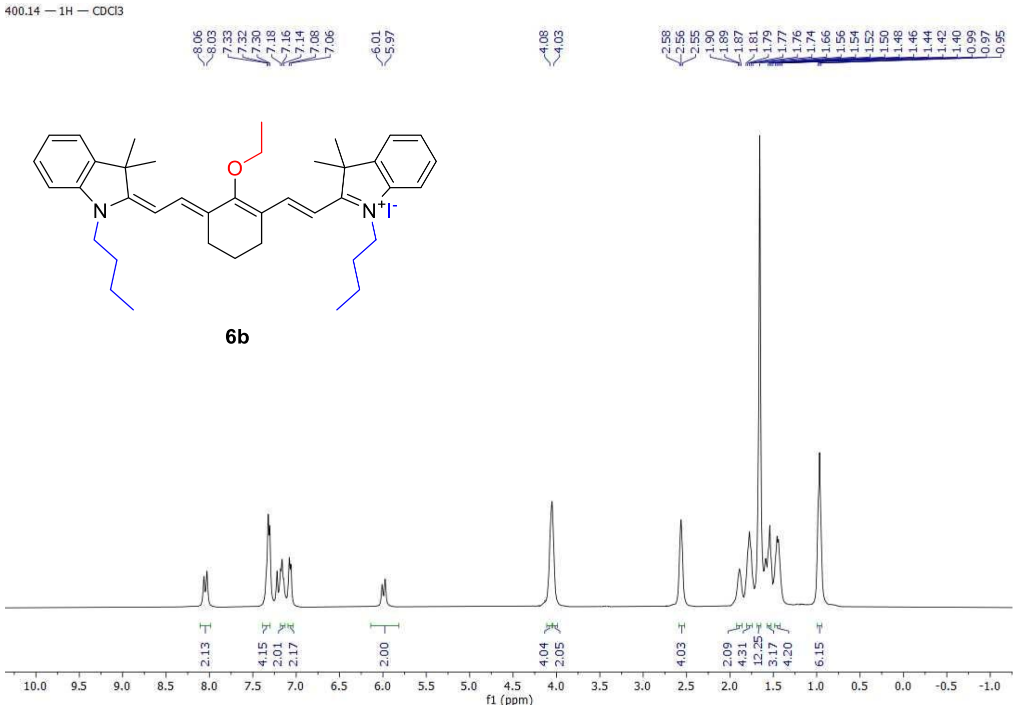

Figure S5. ^{1}HNMR spectrum of fluorophore **6b** in $CDCl_3$ (400 MHz)

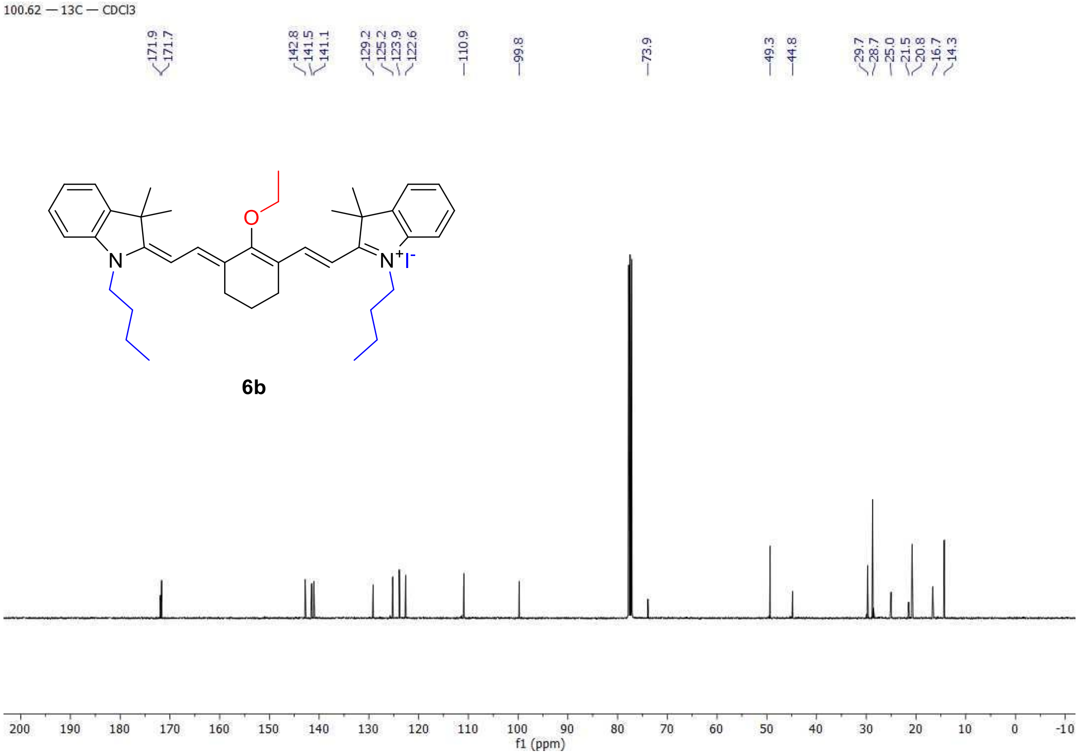

Figure S6. ^{13}CNMR spectrum of fluorophore **6b** in $CDCl_3$ (101 MHz)

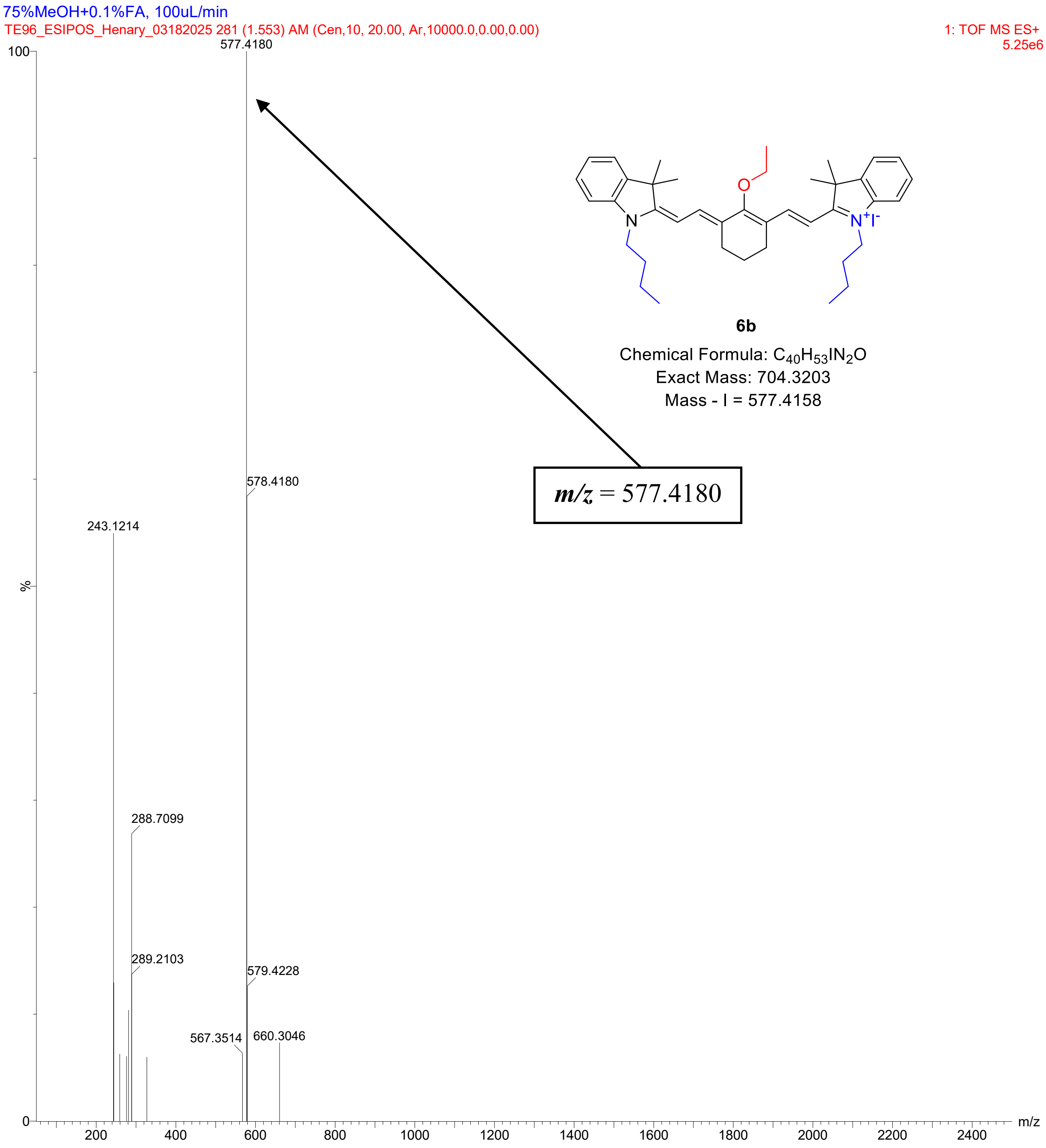

Figure S7. HRMS of fluorophore **6b**

Elemental Composition Report

Single Mass Analysis

Tolerance = 20.0 PPM / DBE: min = -50.0, max = 500.0

Element prediction: Off

Number of isotope peaks used for i-FIT = 3

Monoisotopic Mass, Even Electron Ions

5888 formula(e) evaluated with 1 result within limits (all results (up to 1000) for each mass)

Elements Used:

C: 40-40 H: 0-120 N: 0-20 O: 0-50 Na: 0-2 S: 0-3

Mass	Calc. Mass	mDa	PPM	DBE	i-FIT	Norm		Conf(%)	Formula
Minimum:					-50.0				
Maximum:			1000.0		20.0	500.0			
577.4180	577.4158		2.2	3.8	15.5	38.8	n/a	n/a	C40 H53 N2 O

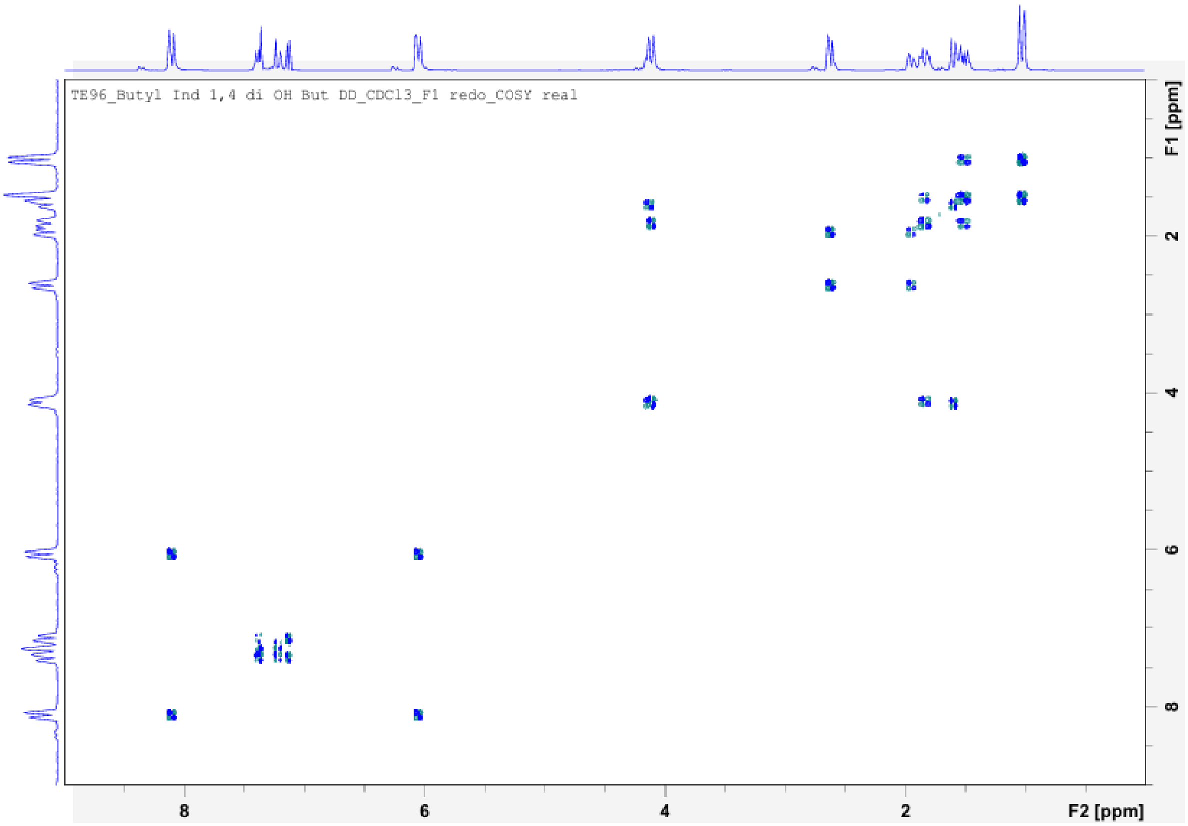

Figure S8. COSY NMR spectrum of fluorophore **6b** in $CDCl_3$ (400 MHz)

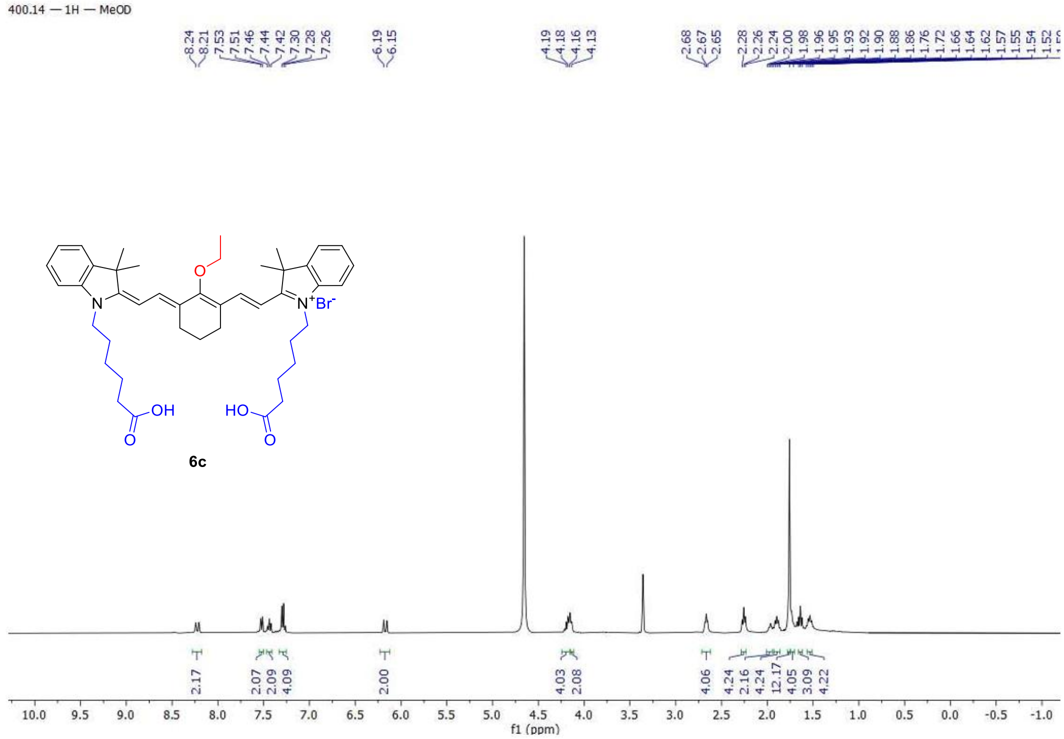

Figure S9. ^{1}HNMR spectrum of fluorophore **6c** in $CDCl_3$ (400 MHz)

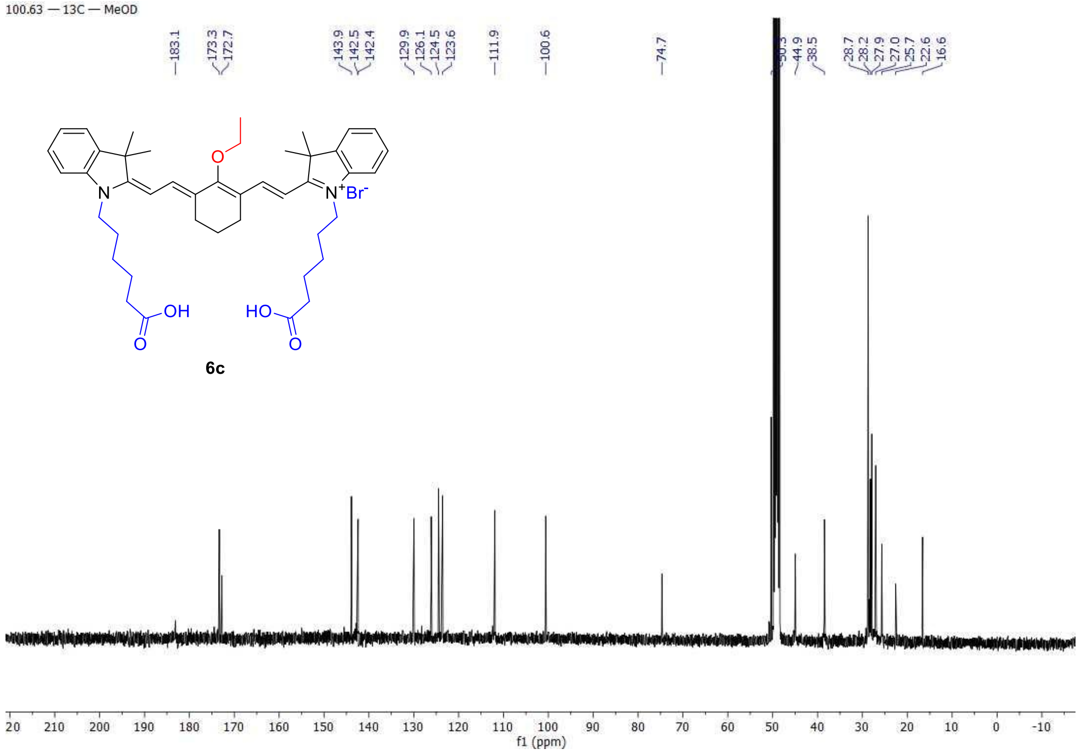

Figure S10. ^{13}CNMR spectrum of fluorophore **6c** in $CDCl_3$ (101 MHz)

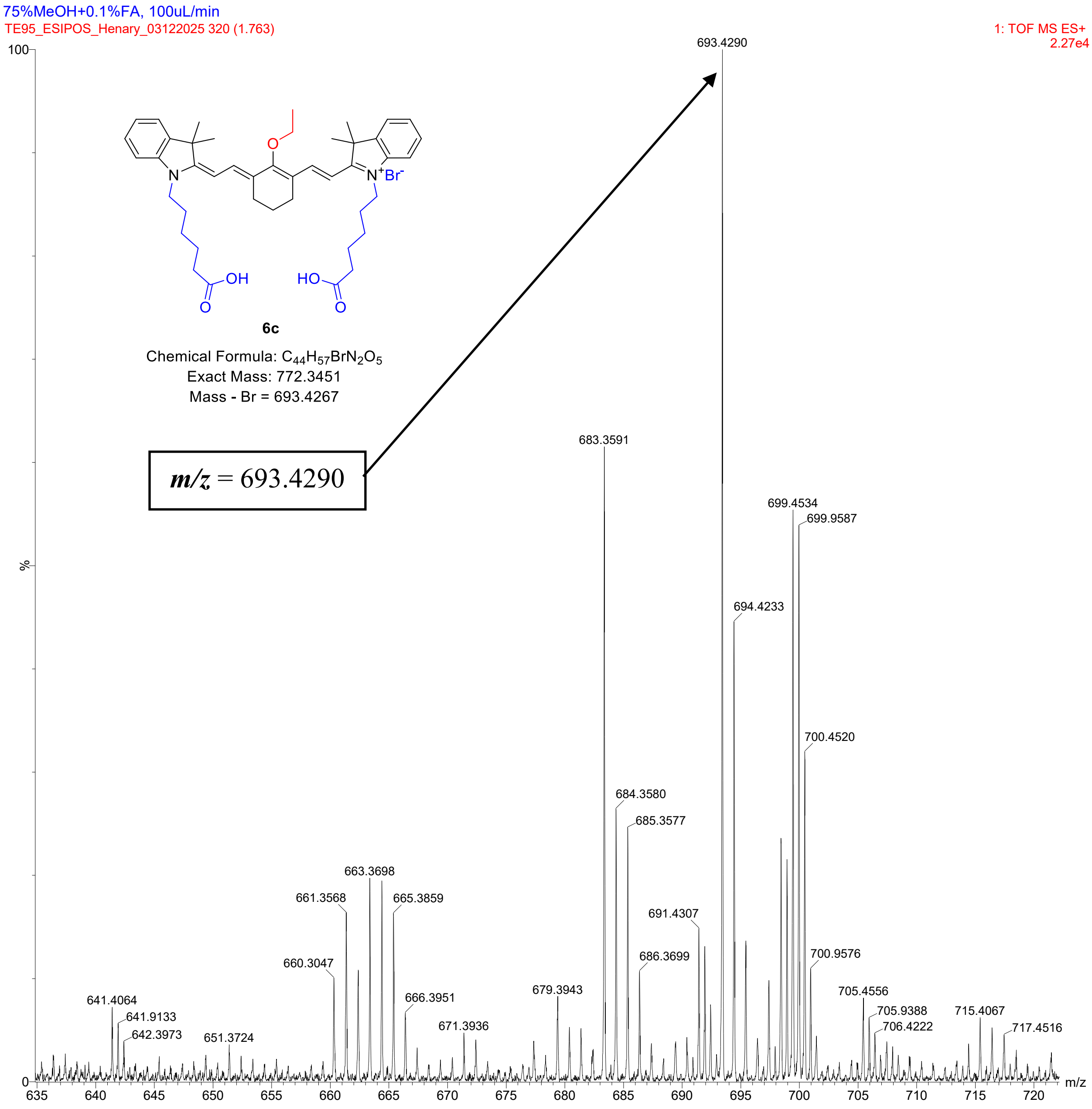

Figure S11. HRMS of fluorophore **6c**

Elemental Composition Report

Single Mass Analysis

Tolerance = 20.0 PPM / DBE: min = -50.0, max = 500.0

Element prediction: Off

Monoisotopic Mass, Even Electron Ions

19388 formula(e) evaluated with 5 results within limits (all results (up to 1000) for each mass)

Elements Used:

C: 44-44 H: 0-120 N: 0-20 O: 0-50 Na: 0-2 S: 0-3 Br: 0-2

Minimum:				-50.0		
Maximum:		1000.0		20.0	500.0	
Mass	Calc. Mass	mDa	PPM		DBE	Formula
693.4290	693.4267	2.3	3.3	17.5	C44 H57 N2 O5	

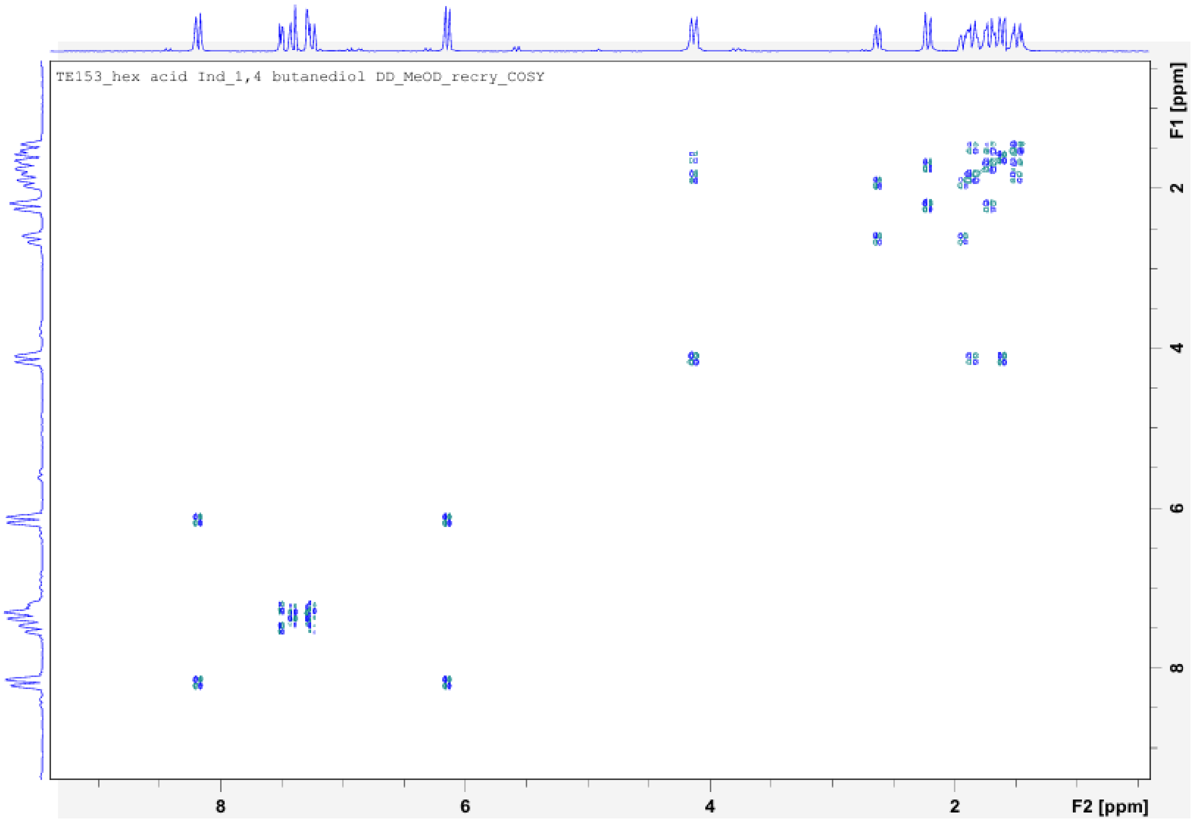

Figure S12. COSY NMR spectrum of fluorophore **6c** in $CDCl_3$ (400 MHz)

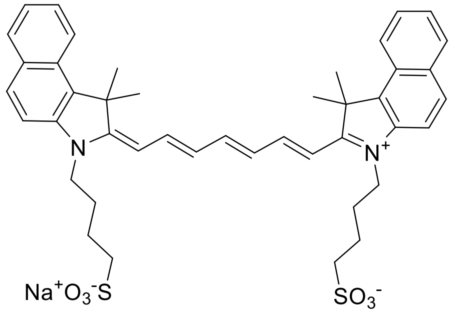

Figure S13. Structure of Indocyanine Green (ICG)

2. Absorbance and Emission Spectra of fluorophores 6a-c

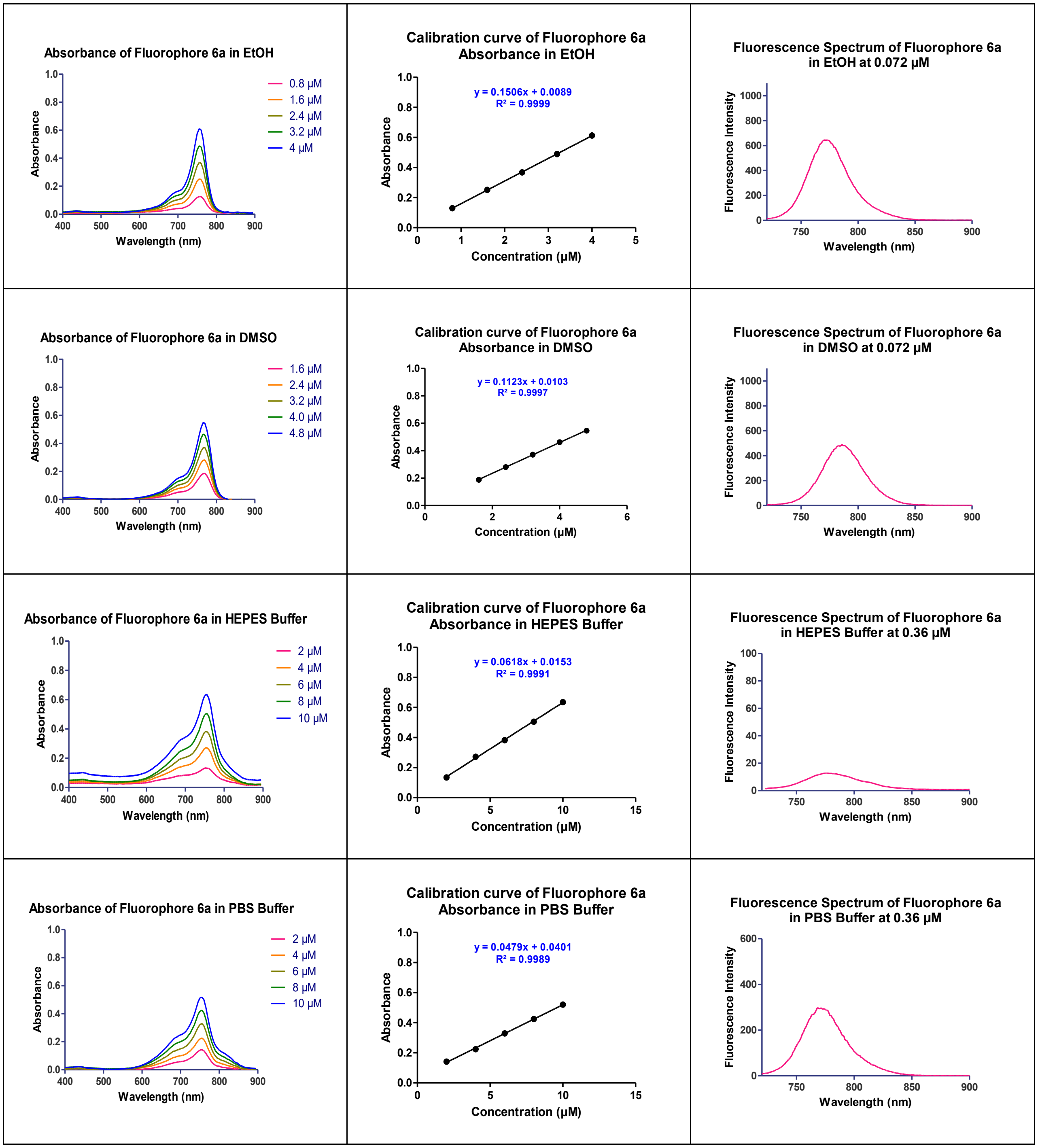

Figure S14. Fluorophore **6a** Absorbance curves at different concentrations, calibration curves, and fluorescence curves (at concentrations of 0.072 to 0.36 μM) in different solvents

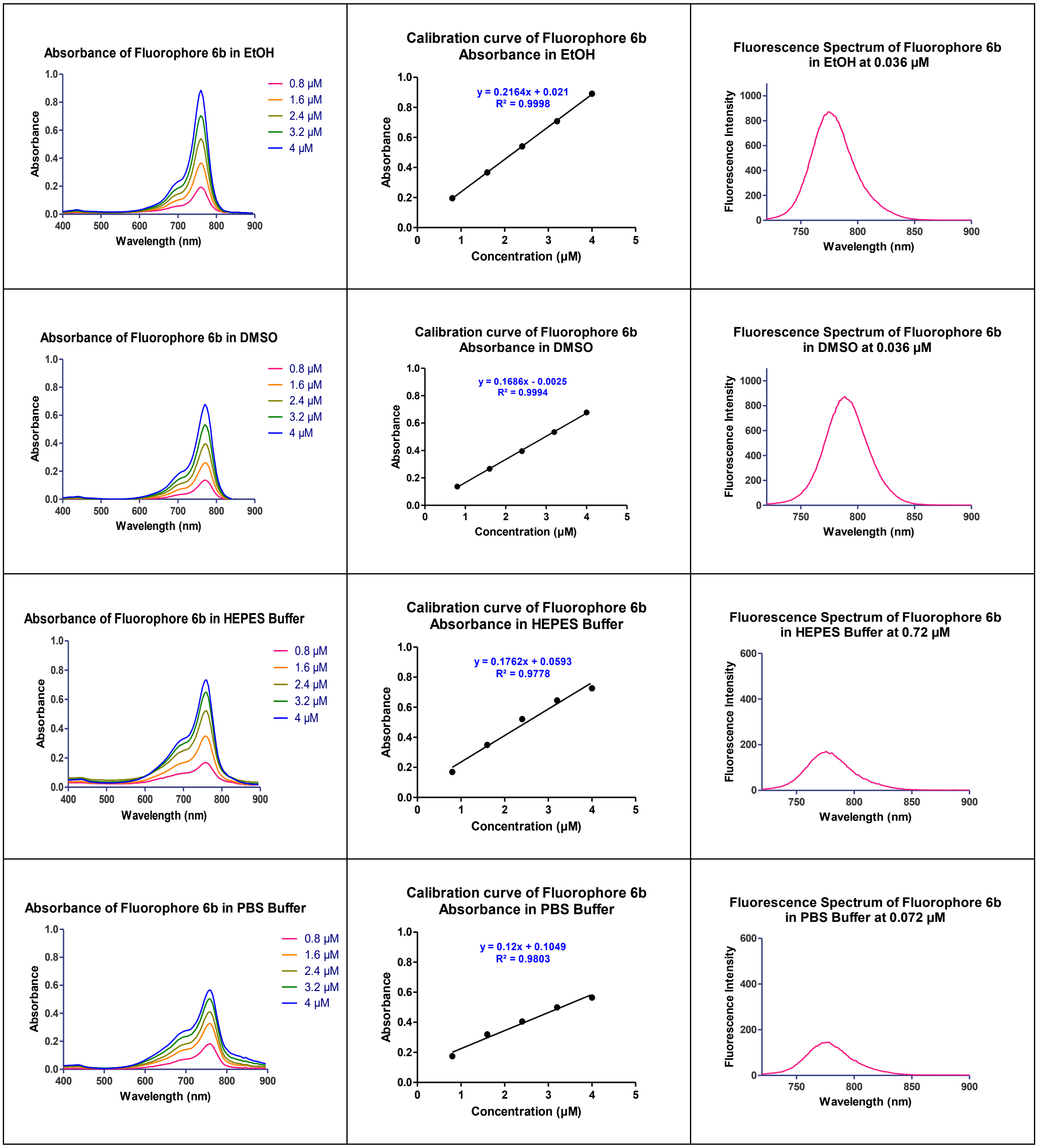

Figure S15. Fluorophore **6b** Absorbance curves at different concentrations, calibration curves, and fluorescence curves (at concentrations of 0.036 to 0.72 μM) in different solvents

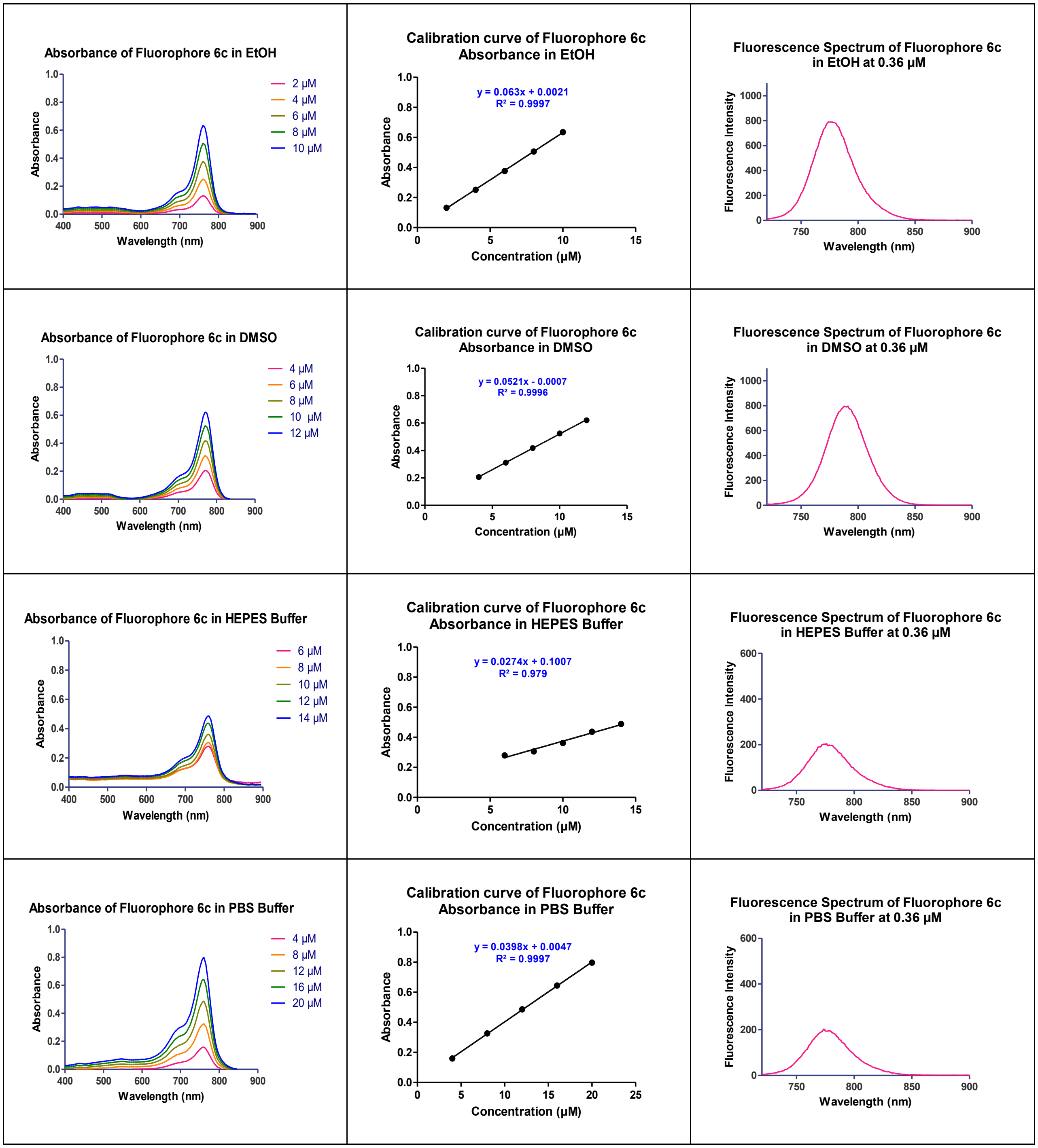

Figure S16. Fluorophore **6c** Absorbance curves at different concentrations, calibration curves, and fluorescence curves (at concentrations of 0.36 μM) in different solvents

3. HOMO and LUMO orbitals of the fluorophores

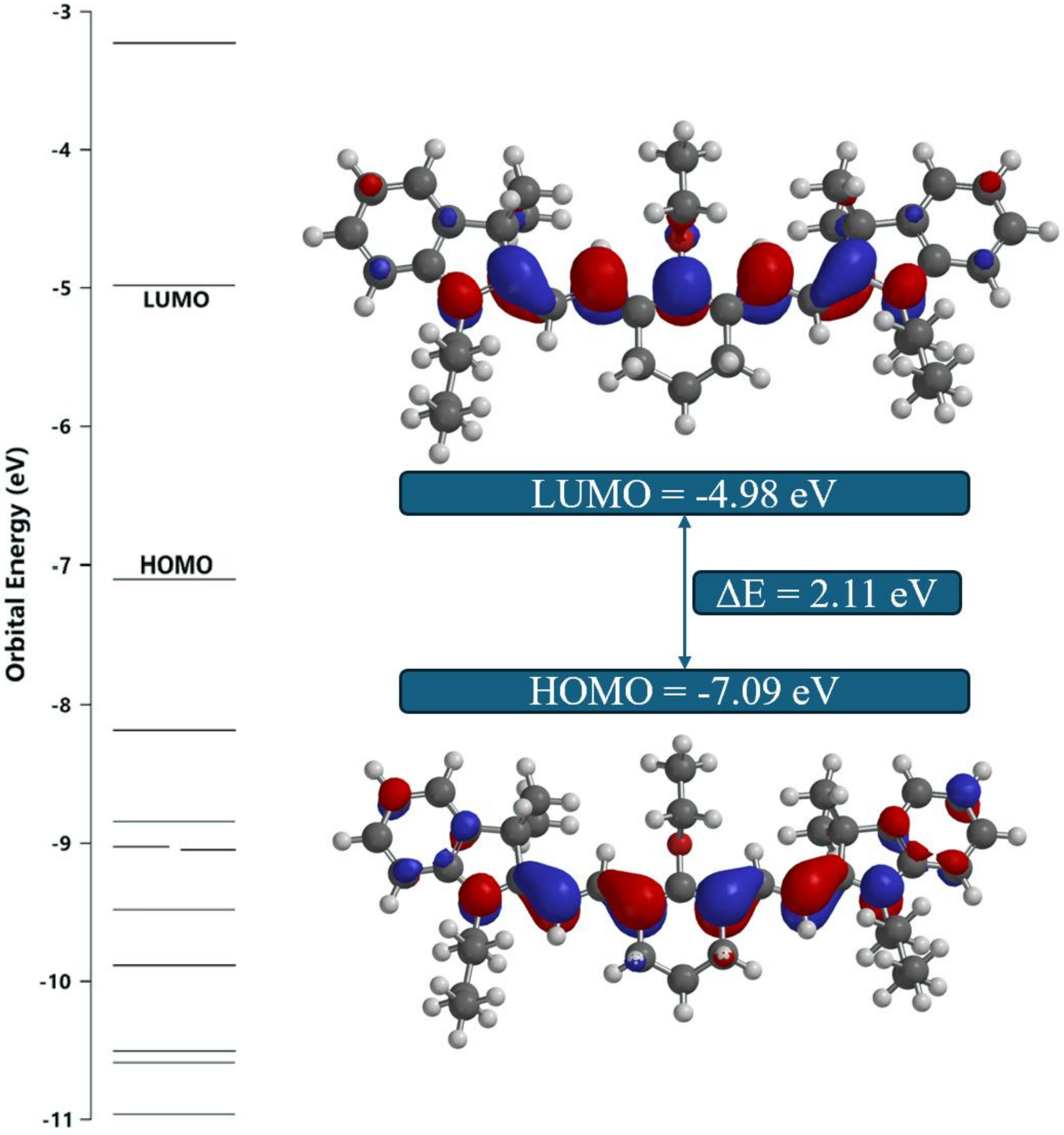

Figure S17. Frontier Molecular Orbitals (HOMO and LUMO) of fluorophore **6b** as predicted by the DFT studies with an energy gap of 2.11 eV

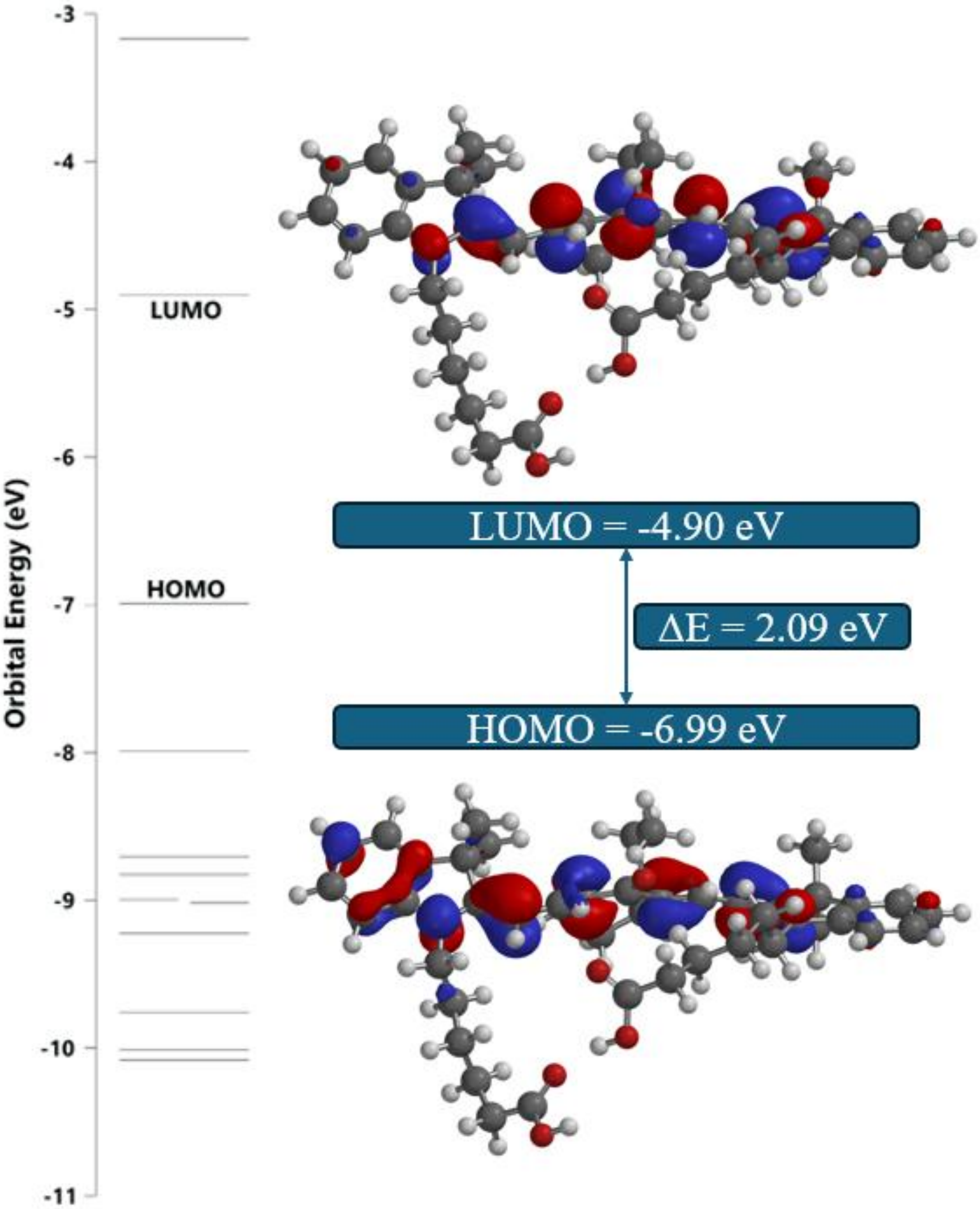

Figure S18. Frontier Molecular Orbitals (HOMO and LUMO) of fluorophore **6c** as predicted by the DFT studies with an energy gap of 2.09 eV

4. Molecular Docking Study

Docking figures of the synthesized fluorophores with bovine serum albumin (BSA)

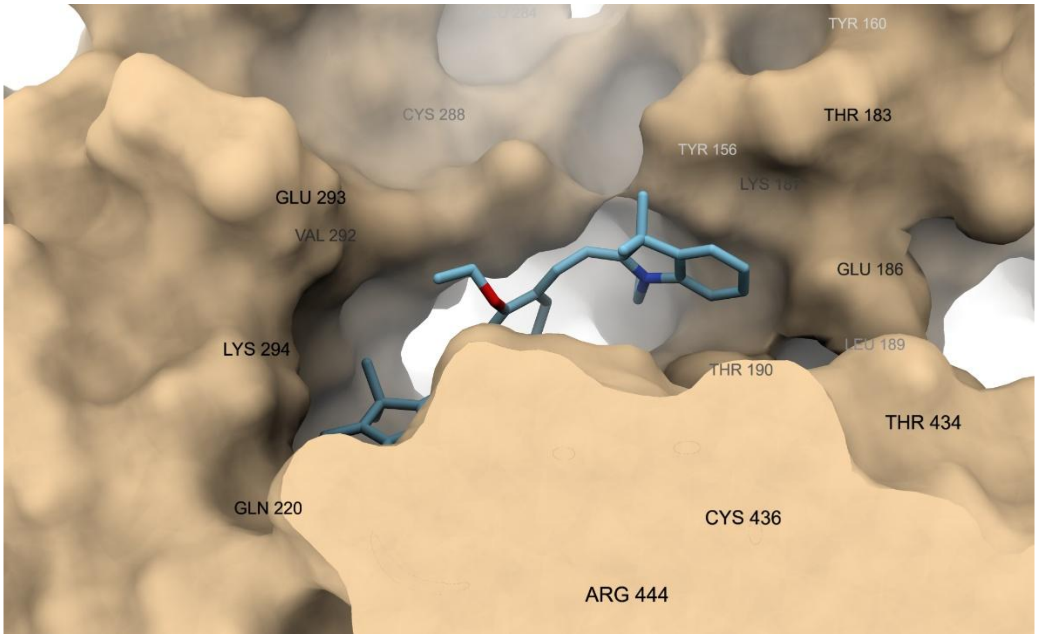

Figure S19. Fluorophore **6a** pose inside bovine serum albumin (BSA, PDB ID: 4jk4)

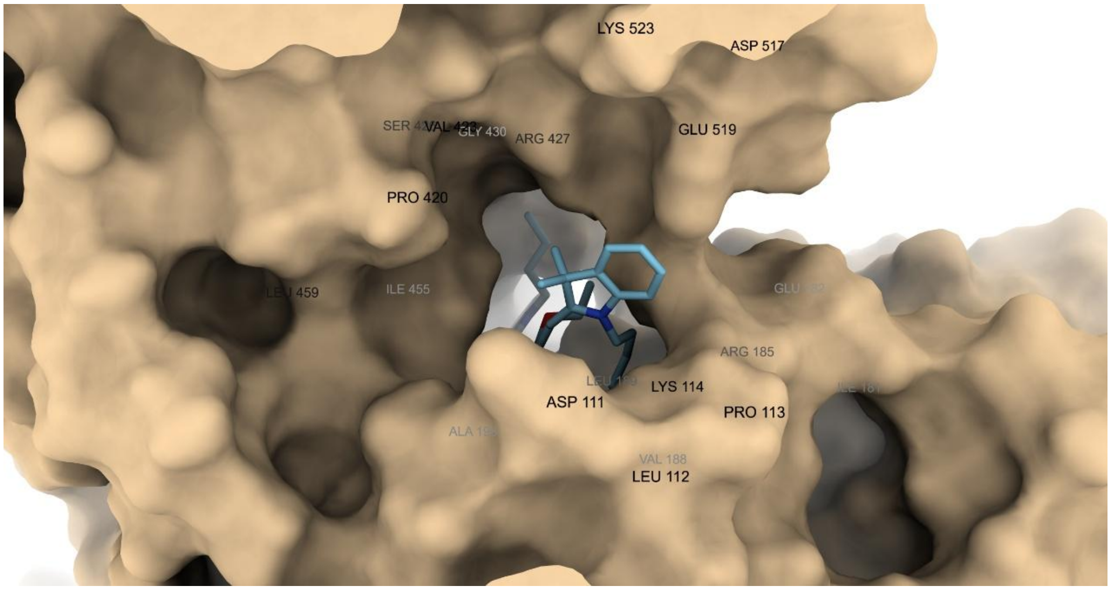

Figure S20. Fluorophore **6b** pose inside bovine serum albumin (BSA, PDB ID: 4jk4)

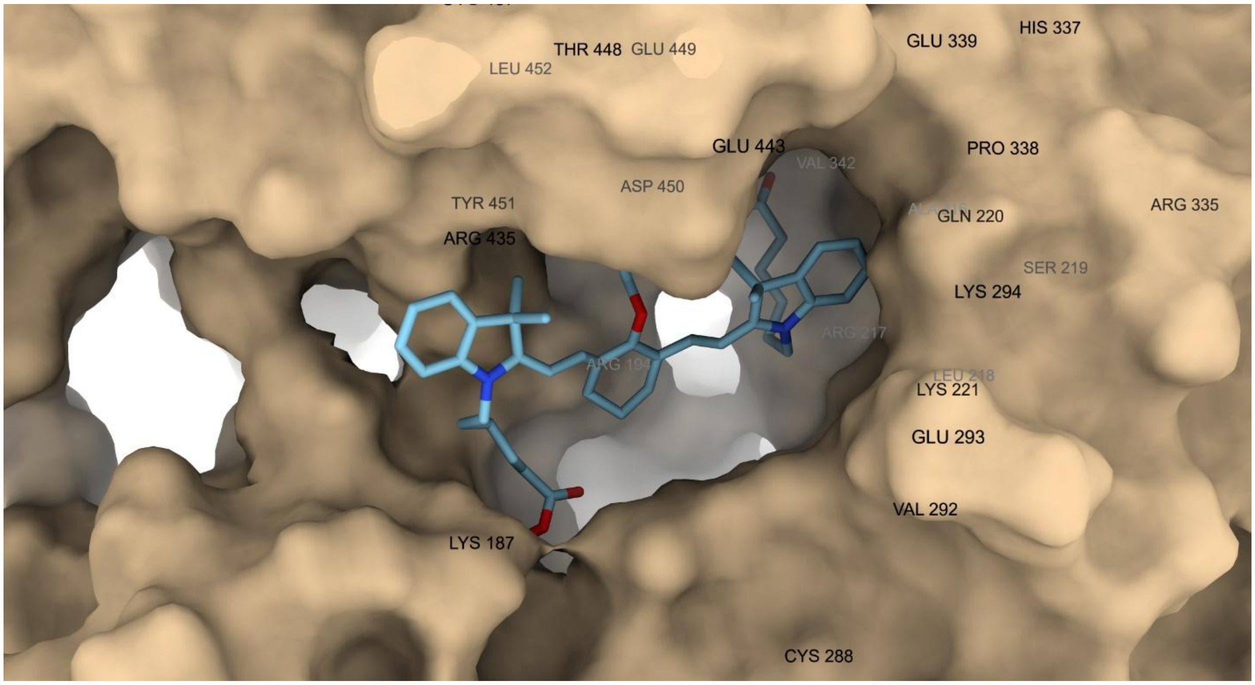

Figure S21. Fluorophore **6c** pose inside bovine serum albumin (BSA, PDB ID: 4jk4)

5. Limit of Detection (LOD) and Limit of Quantitation (LOQ) Calculation

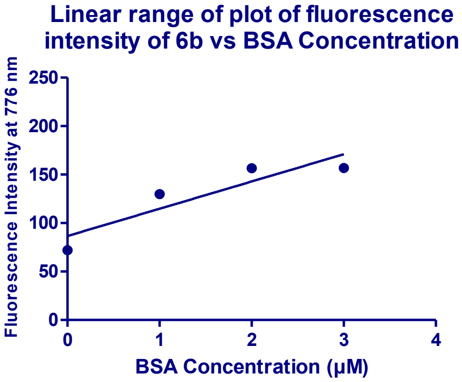

Figure S22. Linear range of fluorescence intensity of fluorophore **6b** vs BSA Concentration for calculation of LOD and LOQ
